# Evaluation of Allelic Expression of Imprinted Genes in Adult Human Blood

**DOI:** 10.1371/journal.pone.0013556

**Published:** 2010-10-21

**Authors:** Jennifer M. Frost, Dave Monk, Taita Stojilkovic-Mikic, Kathryn Woodfine, Lyn S. Chitty, Adele Murrell, Philip Stanier, Gudrun E. Moore

**Affiliations:** 1 Clinical and Molecular Genetics Unit, Institute of Child Health, University College London, London, United Kingdom; 2 Institute of Reproductive and Developmental Biology, Imperial College London, London, United Kingdom; 3 Academic Department of Obstetrics and Gynaecology, University College London, London, United Kingdom; 4 Imprinting and Cancer Group, Cancer Epigenetics and Biology Program, Catalan Institute of Oncology, Hospital Duran I Reynals, Barcelona, Spain; 5 Epigenetics and Imprinting, Cancer Research UK, Cambridge Research Institute, Cambridge, United Kingdom; University of Hong Kong, Hong Kong

## Abstract

**Background:**

Imprinted genes are expressed from only one allele in a parent-of-origin dependent manner. Loss of imprinted (LOI) expression can result in a variety of human disorders and is frequently reported in cancer. Biallelic expression of imprinted genes in adult blood has been suggested as a useful biomarker and is currently being investigated in colorectal cancer. In general, the expression profiles of imprinted genes are well characterised during human and mouse fetal development, but not in human adults.

**Methodology/Principal Findings:**

We investigated quantitative expression of 36 imprinted genes in adult human peripheral blood leukocytes obtained from healthy individuals. Allelic expression was also investigated in B and T lymphocytes and myeloid cells. We found that 21 genes were essentially undetectable in adult blood. Only six genes were demonstrably monoallelic, and most importantly, we found that nine genes were either biallelic or showed variable expression in different individuals. Separated leukocyte populations showed the same expression patterns as whole blood. Differential methylation at each of the imprinting control loci analysed was maintained, including regions that contained biallelically expressed genes. This suggests in some cases methylation has become uncoupled from its role in regulating gene expression.

**Conclusions/Significance:**

We conclude that only a limited set of imprinted genes, including *IGF2* and *SNRPN,* may be useful for LOI cancer biomarker studies. In addition, blood is not a good tissue to use for the discovery of new imprinted genes. Finally, lymphocyte DNA methylation status in the adult may not always be a reliable indicator of monoallelic gene expression.

## Introduction

For the majority of expressed human genes there is both a maternal and a paternal contribution in the offspring. The exception to this is the small subset of imprinted genes that are expressed only from one parental allele or the other. Imprinted genes are important during human development, as demonstrated by the severe syndromes caused by a loss of imprinting (LOI), i.e, the expression of both parental alleles, or the complete loss of expression. Examples include disorders of restricted growth, as seen in Silver-Russell syndrome and over-growth in Beckwith-Wiedemann syndrome, which in most cases result from opposing imprinting disturbances on chromosome 11p15.5 [1]. Imprinting is thought to have evolved in placental mammals as an adaptive mechanism to promote or suppress fetal growth *in utero* [2]. LOI is also seen at high levels (30–100%) in malignant cells in at least 16 different human cancers [3]. At present, LOI is considered to be the most abundant and precocious alteration in cancer [4], [5]. LOI is regarded as a potentially valuable diagnostic tool. Tests based on analysis in adult blood samples of allelic expression of the imprinted *IGF2* gene in the diagnosis of colorectal cancer have become the subject of commercial development [6].

Currently there are approximately 47 protein coding and long, non-coding RNA genes known whose imprinted status in humans has been verified using parent-of-origin *in vivo* expression analyses. In addition, there are a large number of imprinted micro- and SNO RNA molecules (http://igc.otago.ac.nz/). Recently, several genome-wide screens have been carried out, revealing a great many more potentially imprinted transcripts, although these require validation [7]–[10]. To date the majority of confirmed imprinted genes are situated in the genome in clusters. This genomic organisation allows their expression to be collectively regulated by a nearby imprinting control region (ICR) or differentially methylated region (DMR), which is methylated on one allele but not the other. This methylation is established in the germline during gonad development of the previous generation and controls the allele specific expression [11]. The imprinted expression of some genes is restricted to specific tissues, and is either: not expressed at all elsewhere e.g. *PHLDA2*, whose expression is limited to the placenta [12]; expressed biallelically elsewhere e.g. *GNAS* which in the human is ubiquitously expressed but imprinted only in the thyroid, ovary and pituitary gland [13]; or expressed from the opposite allele in different tissues e.g. *GRB10* which is paternally expressed in brain but maternally expressed in the placenta [14]. For other genes, imprinted expression is found in the majority of tissues during fetal development, as in the case of *IGF2*, but becomes biallelic in some tissues in the adult [15]. Regulation of this temporal and tissue specificity is likely to be brought about through transcription factor binding to alternative promoters and enhancers. Once expression is enabled, imprinting cluster-specific mechanisms promote or prevent transcription of individual alleles. This control may include antisense transcription of non-coding RNAs, allelic histone modification, post-zygotic DNA methylation, RNA interference and the blocking of enhancers by repressors of boundary elements/transcription factors such as CTCF and YY1 [16].

Imprinted gene expression status in the healthy adult human has for obvious reasons been difficult to characterise. Peripheral blood leukocytes (PBL) are unique as they provide a practical and available resource for both genomic and epigenomic human analyses. Imprinting expression patterns and related mechanisms in the human have largely been extrapolated from experiments in the mouse, although it is clear that there are important differences between the two [11], [17]. To fill this knowledge gap, particularly with the growing importance of LOI as a marker in cancer diagnostics, this study was designed to investigate genomic imprinting in adult human PBL, analysing 36 gene transcripts, including representatives from all of the known human imprinted cluster loci and several isolated imprinted genes. Expression of the 36 transcripts was quantified in adult PBL and in normal first trimester placenta, liver and brain. As three of the most important fetal organs for growth and development, these tissues express key imprinted genes at high levels [12], [18] and are also representative as sites of tissue-specific expression [19]. To investigate the actual imprinting status of the genes expressed in adult blood, we genotyped common expressed single nucleotide polymorphisms (SNPs) in a cohort of 50 healthy adults and analysed allelic expression in both whole blood and fractionated cellular sub-populations. Finally, DNA methylation at ICRs was measured in order to verify DMR maintenance and potentially identify the source of any imprinting errors.

## Materials and Methods

### Ethics Statement

Ethical approval for adult blood and fetal tissue collection was granted by Hammersmith, Queen Charlotte's & Chelsea and Acton Hospitals Research Ethics Committee, Project Registration Nos: 2001/6029 and 2001/6028, respectively.

### Participants

Adult volunteers (n = 50) were randomly selected with a male:female ratio of approximately 1∶1, between the ages of 20 to 50 years old, and included individuals of different ethnicity. Fetal tissues were obtained from first trimester products of conception from a normal white European cohort.

### Sample preparation

Peripheral blood leukocytes (PBL) were extracted from 15 ml fresh whole blood using Lymphoprep™ (Axis Shield, Norway) according to the manufacturer's instructions. Genomic DNA was purified by standard phenol-chloroform extraction. For lineage-specific analysis, whole blood was separated into CD3, CD15 and CD19 positive cell fractions using an autoMACS™ Separator (Miltenyi Biotech, Surrey UK). Total RNA was extracted using Trizol (Gibco BRL, Paisley, UK), and treated with TURBO DNase (Ambion, Cambridgeshire, UK) and DNAse1 (Invitrogen, Paisley, UK). RNA was randomly primed and reverse-transcribed using Moloney murine leukemia virus reverse transcriptase (MMLM RT; Promega, Southampton UK). Parallel control samples omitting MMLV RT were generated in each case. Adult bone marrow RNA was purchased from Clontech Laboratories Inc (CA). *KCNQ1OT1* strand specific cDNA was created in the same way except *B-ACTIN* reverse and *KCNQ1OT1* reverse primers were used instead of random primers.

### Non-quantitative RT-PCR

Cycle profiles for a mixed fetal tissue cDNA sample and a mixed PBL sample (n = 50) were carried out using non-quantitative (nq)RT-PCR for all genes and resolved on 1.5% agarose gels stained with ethidium bromide. Gel quantitation was performed using Imagemaster VDS 2.0 (Pharmacia Biotech, Bucks, UK) and amplicon densitometry readings vs cycle plots generated for each gene (data not shown). Mid log-linear phase cycle numbers were then used for allelic expression nqRT-PCR (Table S1).

### Quantitative expression of imprinted genes in blood

Total RNA was isolated from two indidvidual adult PBL samples, second trimester fetal brain, placenta and liver samples (each pooled from three fetuses) and adult bone marrow (from six individuals aged 38–59 years). cDNA amplification was quantitated with SYBR green dye using the Applied Biosystems 7500 Fast Real-Time PCR System (Cheshire, UK) for 40 cycles. Delta CTs (ΔCTs) were generated in triplicate of each transcript using *GAPDH* as an endogenous control. Primer sequences and conditions are listed in Table S2.

### Allelic expression analysis

Genotypes were obtained for expressed genes in 50 individuals using a minimum of one exonic SNP for each gene either by digestion or sequencing. Sequences were interrogated using Sequencher™ v4.6 (Gene Codes Corporation, MI) to distinguish heterozygotes (informative) and homozygotes. Informative samples were analysed for allelic expression using intron crossing primers (Table S3). Sense/antisense pairs (*KCNQ1/KCNQ1OT1* and *MEST/MESTIT1*) were analysed independently by ensuring antisense transcript amplicons lay wholly within introns of the sense genes. In addition, the samples informative for *KCNQ1OT1* were analysed using strand specific RT-PCR. cDNA was prepared using only *B-ACTIN* and *KCNQ1OT1* reverse oligonucleotides and RT-PCR carried out as before. For lineage-specific analysis, whole blood from three individuals was separated into CD19+ B-lymphocytes (5% total cell count), CD3+ T-lymphocytes (25%) and CD15+ myeloid cells (50–70%) using an autoMACS™ Separator (Miltenyi Biotech, Surrey UK).

No parental genotypes were included so imprinting is inferred from monoallelic expression in heterozygous individuals. Primer sequences and conditions for genotyping and allelic expression analysis are listed in Table S3.

### Analysis of differentially methylated regions

Combined bisulphite and restriction analysis (CoBRA; Xiong and Laird, 1997) was performed using sodium bisulphite treated PBL DNA samples (n = 2) and purified using the EZ Gold Methylation Kit™ (ZYMO, Orange, CA). Bisulphite specific primers (Table S4) for each ICR were used with Hotstar Taq polymerase (Qiagen, West Sussex, UK) at 45 PCR cycles and amplicons digested using either *Taqα*1, *Tai*1, *Mbo*I or *Bst*UI restriction enzymes (enzymes used for each amplicon shown in Table S4; New England Biolabs). Differentially methylated products were resolved on 3–4% agarose gels and stained with ethidium bromide. For pyrosequencing analysis, each assay included three to eleven CpGs, 12 individuals were analysed. Genomic DNA was bisulphite treated using EZ DNA methylation kit (Zymo Research) and used to generate PCR amplified templates in a two round PCR. The first round used unique forward and reverse primers. The reverse primer contains an M13 sequence, which was used to tag the 5′ end with biotin prior to sequencing (specific primers and PCR conditions are available on request). Pyrosequencing was carried out on PSQ HS 96 System and PyroMark MD System using Pyro Gold Reagent kits (Biotage, Uppsala, Sweden). Methylation was quantified using Pyro Q-CpG Software (Biotage, Uppsala, Sweden) that calculates the ratio of converted C's (T's) to unconverted C's at each CpG and expresses this as a percentage methylation. Average methylation across the DMR regions for all CpGs was calculated. Median and IQR methylation for different samples were analysed using Prism Graphpad software.

## Results

### Analysis of imprinted gene expression levels in adult PBL

**Figure 1:**
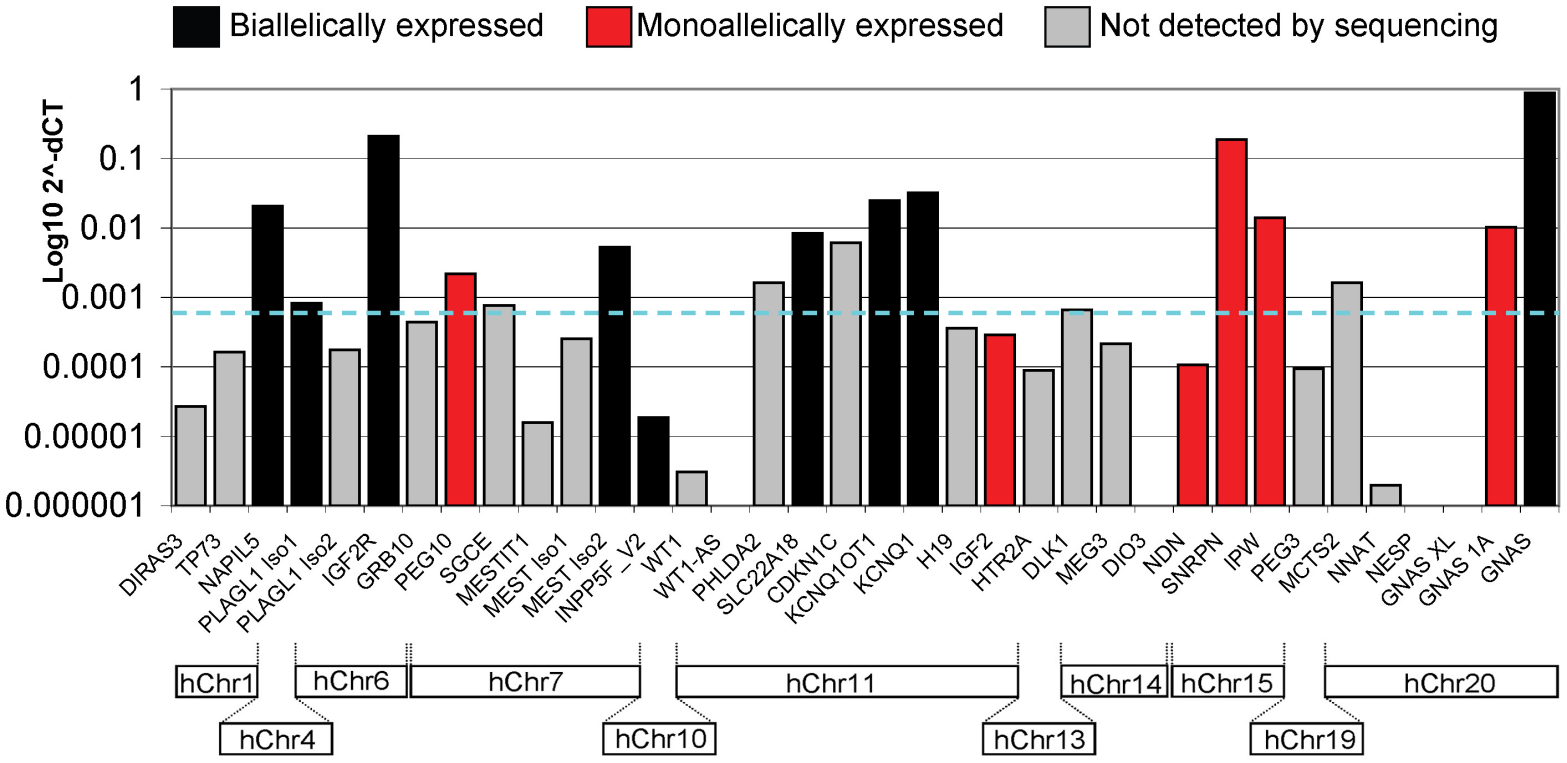
Quantitative expression levels and allelic status of imprinted genes in the PBL of two individuals. (i) Allelic expression of the transcripts we could detect with non-quantitative RT-PCR is shown, aligned with the genes from bar graph (ii). Red bars represent monoallelic expression and black bars biallelic expression. Using a maximum of 45 cycles the transcripts we could amplify predominately corresponded to those which on Q-PCR had a ΔCT <9, shown by the dotted line. The grey bars represent transcripts that could not be detected using non-quantitative RT-PCR methods so could not be assigned an allelic preference. (ii) Gene names are plotted on the x axis, against log(10) 2∧-ΔCt (ΔCt  =  the cycle number at which transcript level reached threshold, corrected to an endogenous control) on the y, using thresholds defined by the linear phase of curves generated from the real-time amplification. ΔCTs for triplicates of each transcript were calculated using a *GAPDH* endogenous control, thus log(10) 2∧-ΔCt of *GAPDH*  = 1. (iii) Outline of the position of each gene within the human genome, demonstrating the extent of each imprinting cluster.

In order to study imprinting in adult blood, we first investigated the mRNA expression levels of 36 gene transcripts by quantitative (q)RT-PCR (Fig. 1), which was performed in two unrelated healthy adult individuals. Expression values were based on the amplified product reaching a gene expression threshold (T) following a number of PCR cycles (C), normalised (Δ) to the expression of an endogenous control (*GAPDH*). The ΔCT value was then used as a measure of relative expression. Gene expression between the two individuals showed a high level of consistency, where ΔCT of each gene for the two individuals differed by two PCR cycles or less for 81% of the genes analysed. Of these, 50% had a difference of only one cycle between the two individuals (Table S1).

Non-quantitative (nq-)RT-PCR was carried out in parallel to qRT-PCR in order to analyse allelic expression on a pool of the PBL cDNA from our 50 individuals. NqRT-PCR was able to generate amplicons that could be reliably analysed by sequencing when the qRT-PCR ΔCT for that gene was below nine in the majority of cases. A ΔCT value of nine is equivalent to 2^−^Δ^CT^ of 0.0015, and this is indicated on the graph in Fig. 1 by a dotted line. A ΔCT of above nine generally correlated with gene expression that was too low to be amplified by nqRT-PCR and sequenced.

Three genes were expressed at notably high levels: *GNAS*, *SNRPN* and *IGF2R.* Another nine genes, *KCNQ1, KCNQ1OT1, NAP1L5, IPW, GNAS EXON 1A, SLC22A18, CDKN1C, MEST ISOFORM 2* and *PEG10* were expressed at a level decreased by approximately ten fold relative to the three highly expressed genes, and the remaining 24 genes decreased by at least 100 fold. In comparison to PBL, fetal placenta, liver and brain expression levels were higher both in magnitude and in representative coverage for 28 of the 36 analysed transcripts. These data indicate that overall, compared to fetal tissues, imprinted genes are not highly expressed in adult blood (Fig. S1). There were eight genes whose expression levels in PBL were comparable to or increased compared to the other tissues; *NAP1L5, PLAGL1* Isoform 2, *IGF2R, SLC22A18, KCNQ1OT1, KCNQ1, IPW*, *SNRPN, TP73* and *GNAS* (Fig. S1).

### Analysis of allelic expression status of imprinted genes in adult PBL

**Figure 2:**
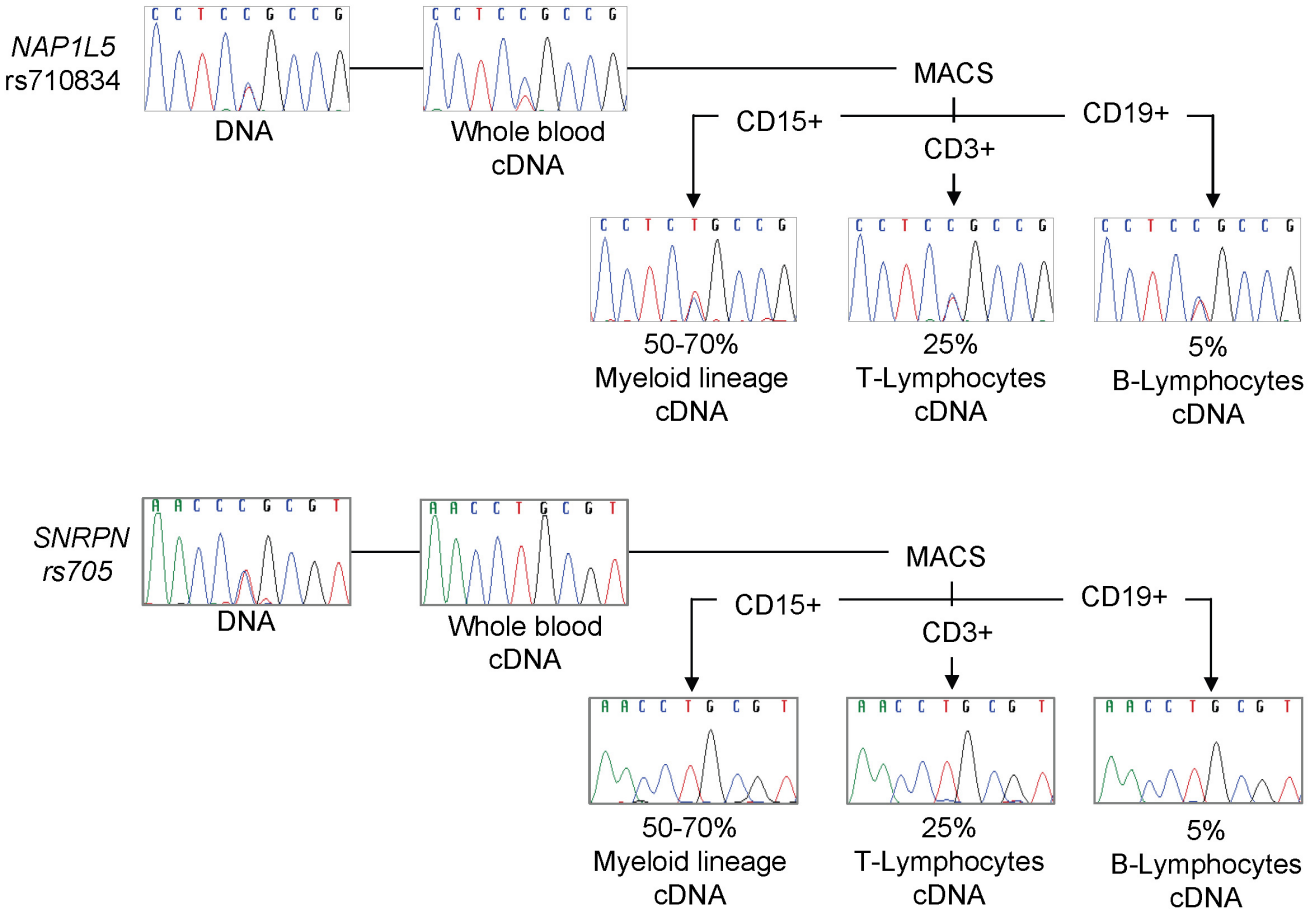
Allelic expression analysis of whole blood and immune-separated leukocyte fractions. Exemplar sequence traces of RT-PCR amplicons represent the genotypes and expression at specific SNPs for (i) *NAP1L5* and (ii) *SNRPN*, in whole blood peripheral leukocytes, and then in each separated lineage component. It is clear that the lack of imprinting for *NAP1L5* seen in the fractions containing unseparated PBL, or indeed the maintained imprinting of *SNRPN*, is not due to either allele switching or the masking of underlying imprinted expression in a cell subtype, at least for the cell populations examined here. Table 2 lists the complete data set of allelic expression of imprinted genes in separated PBL cell populations from three individuals.

To investigate the actual imprinting status of genes in adult blood, we genotyped 50 unrelated individuals with respect to common exonic SNPs present in the 15 transcripts that were expressed at sufficient levels for detection by sequencing. This analysis revealed three groups of genes (Fig. 1 and Table 1): Group 1: *PLAG1* Isoform 2, *IGF2R, MEST* Isoform 2 (polymorphic), *KCNQ1* and *GNAS* were expressed biallelically. Each of these genes are widely expressed but imprinted only in certain tissues, or are developmentally regulated, so their biallelic status in PBL was not surprising; Group 2: *PEG10, IGF2, SNRPN, NDN, IPW* and *GNAS EXON 1A* were expressed monoallelically, which was consistent with their reported status in human fetal and adult tissues; and finally Group 3: *NAP1L5, INPP5F_V2* (polymorphic), *SLC22A18* and *KCNQ1OT1* which were expressed biallelically. An apparent allelic bias was present in some of the samples for *NAP1L5* (Fig. 2), although without parental DNA we cannot establish which allele was preferentially expressed. This bias was not present upon separation of the whole blood into individual fractions. Strand-specific RT-PCR of *KCNQ1OT1* transcripts was carried out to ensure no unspliced nascent *KCNQ1* transcripts were amplified, and also showed *KCNQ1OT1* to be biallelic (Fig. S2). Group 3 is distinct from Group 1 as the biallelic expression of the genes in Group 3 was unexpected given their monoallelic expression status in other tissues. Interestingly, of the ten genes that were more highly expressed in blood than fetal tissues (Fig. S1), only *SNRPN* remained monoallelic (IPW and TP73 could not be analysed as they were undetectable by nqRT-PCR).

**Table 1:** Summary of expression levels and allele specificity in human PBL.

Locus/Cluster	Transcripts in cluster & accession	UCSC SNP	Methylated DMR*	P of O**	MeanΔCT	QPCR relative exp	RT PCR	Allelic Expression
4q22 *NAP1L5*	*NAP1L5:* NM_153757	rs710834	Prom MAT^G^	Pat	5.6	Highest	33	9/9 B
6q24 *PLAGL1*	*PLAGL1* Isoform 2: NM_001080951	rs9373409	Prom MAT^G^	B	12.6	Highest	45	3/3 B
6q25 *IGF2R*	*IGF2R:* NM_000876	rs614754		Mat	2.3	Highest	35	1/1 B
7q21 *PEG10*	*PEG10* NM_015068	*rs13073*	Prom MAT^G^	Pat	9.1	Pl, Li, Br	36	4/4 M
7q32 *MEST*	*MEST* Iso 2 NM_177524			Pat	7.6	Br	35	8/9 B; 1/9 M
10q26	*INPP5F_V2:* NR_003252	*rs3188055*	Prom MAT^G^	Pat	16.0	Pl, Li, Br	40	8/11 M; 3/11 B
11p15 *KCNQ1*	*SLC22A18* NM_183233	*rs1048046/7*	KvDMR MAT^G^	Mat	8.2	Highest	40	5/5 B
	*CDKN1C* NM_000076	PAPA RPT		Mat	7.5	Pl	U	U
	*KCNQ1OT1* NM_000128	*rs231357/9*		Pat	5.4	Highest	40	6/6 B
	*KCNQ1* NM_000218	*rs1057128*		Mat	5.0	Highest	38	3/3 B
11p15 *IGF2/H19*	*IGF2* NM_000612	*rs680*	DMR0,1,2 PAT^S^ PAT^G^ PAT^S^	Pat	11.8	Pl, Li, Br, BM	40	20/20 M
15q11 *SNURF/SNRPN*	*NDN* NM_002487	*rs2192206*	Prom MAT^?^	Pat	13.6	Pl, Li, Br, BM	40	3/3 M
	*SNRPN* NM_022806	*rs705*	Prom MAT^G^	Pat	2.4	Highest	40	3/3 M
	*IPW* NR_023915	*rs691*		Pat	6.2	Br, Li	35	5/5M
20q13 *GNAS*	*GNAS* NM_000516	*rs7121*	1A Prom MAT^G^	Mat	0.3	Highest	33	13/13 B
	*Exon 1A* X56009		1A Prom MAT^G^	Pat	6.7	Pl	40	7/7 M

**Cluster/Locus -** the cluster where the transcripts analysed **(Transcripts in cluster & accession)** are located. **UCSC SNP annotation** - the expressed polymorphism analysed for the allelic expression data. **Methylated DMR** - the differentially methylated regions associated with each cluster and the parental origin of the methylated allele,

*DMR identified in previously published work from other laboratories (see Table S1 for references); Prom  =  promoter; MAT/PAT  =  maternal/paternal allele methylated; ^G/S^  =  DMR established in the germline/post fertilisation. **P of O** – the parental origin of the expressed allele

**established in previously published work from other laboratories – extensive references available for each gene @ http://igc.otago.ac.nz/table.html. Mat/Pat  =  maternal/paternal allele expressed. **Mean** Δ**CT 1d.p**. - the average of ΔCT values of PCL samples A and B for each gene to one decimal place. Generally ΔCT values above 9 ( = 2∧-dCT of 0.001) could be deteced by non-quantitative (nq) RT-PCR. U  =  below nq RT-PCR detection. The separate Q-PCR expression data from each individual are shown in Table S1. **QPCR relative exp** - the expression of these genes relative to the other tissues examined in Fig. S1. Pl  =  Placenta; Li  =  Liver; Br  =  brain; BM  =  bone marrow. Genes which were most highly expressed in PBL compared to fetal tissues are denoted ‘Highest’; in all cases except for *SNRPN*, these genes are biallelically expressed. **RT-PCR** - the number of nq RT-PCR cycles required to generate a product that could be sequenced reliably. The fractions in the **Allelic Expression** column indicate the number of biallelic or monoallelic samples there were of the total that were heterozygous (i.e. informative) for each SNP. The number of informative samples represents the total found following genotyping of 50 individuals., M/B  =  mono/biallelic. The complete data set and references, (data from humans and mice) can be found in Table S1.

**Table 2:** Expression and allele specificity in separated myeloid, T and B lymphocyte fractions.

Gene	n	Whole PBL	Myeloid Cells	T-Lymphocytes	B-Lymphocytes
*NAP1L5*	1	Biallelic	Biallelic	Biallelic	Biallelic
*PLAGL1 Iso1*	1	NE	NE	Monoallelic	Monoallelic
*PLAGL1 Iso2*	1	Biallelic	Biallelic	Biallelic	Biallelic
*MEST Iso2*	1	Biallelic	Biallelic	NE	Biallelic
*KCNQ1OT1*	2	Biallelic	Biallelic	Biallelic	Biallelic
*SLC22A18*	2	Biallelic	Biallelic	Biallelic	Biallelic
*IGF2*	1	Monoallelic	NE	Monoallelic	NE
*SNRPN*	1	Monoallelic	Monoallelic	Monoallelic	Monoallelic
*NDN*	2	Monoallelic	Monoallelic	Monoallelic	Monoallelic

M, monoallelic; B, biallelic; NE, not expressed. Three informative individuals were selected, and n numbers are the number of individuals informative for SNP in each respective gene. Expression patterns varied slightly but allele specificity was identical between sub-populations.

PBL are a circulating cell population, and are therefore not in contact with a static cell niche. To evaluate changes in gene expression from the time of cell release into the circulation, we also analysed adult human bone marrow samples (n = 6), as the origin of the predominant myeloid cell population in peripheral blood (Fig. S1). Although displaying the same pattern of expression where *GNAS, SNRPN* and *IGF2R* are most highly expressed, bone marrow has a lower level of expression of imprinted genes compared to PBL (Tables 1 and S1).

### Analysis of allelic expression in separated leukocyte populations

Both imprinted and non-imprinted monoallelic expression can be cell-type specific within certain organs [20], [21]. To ensure that a sub-population of biallelically expressing cells was not masking monoallelic expression from other blood lineages we isolated the three predominant cell types found in the PBL population. PBL samples from three informative individuals were analysed with respect to the allelic expression of *NAPIL5, PLAGL1* Isoform 1, *PLAGL1* Isoform 2, *MEST* Isoform 2, *KCNQ1OT1, SLC22A18, IGF2, SNRPN* and *NDN* in CD19+ B-lymphocytes (5%), CD3+ T-lymphocytes (25%) and CD15+ myeloid cells (50–70%). The biallelic/monoallelic expression pattern of the genes was identical for each cell type and for the total PBL population previously analysed (Fig. 2 and Table 2).

### Analysis of differential methylation at imprinting control regions

**Figure 3:**
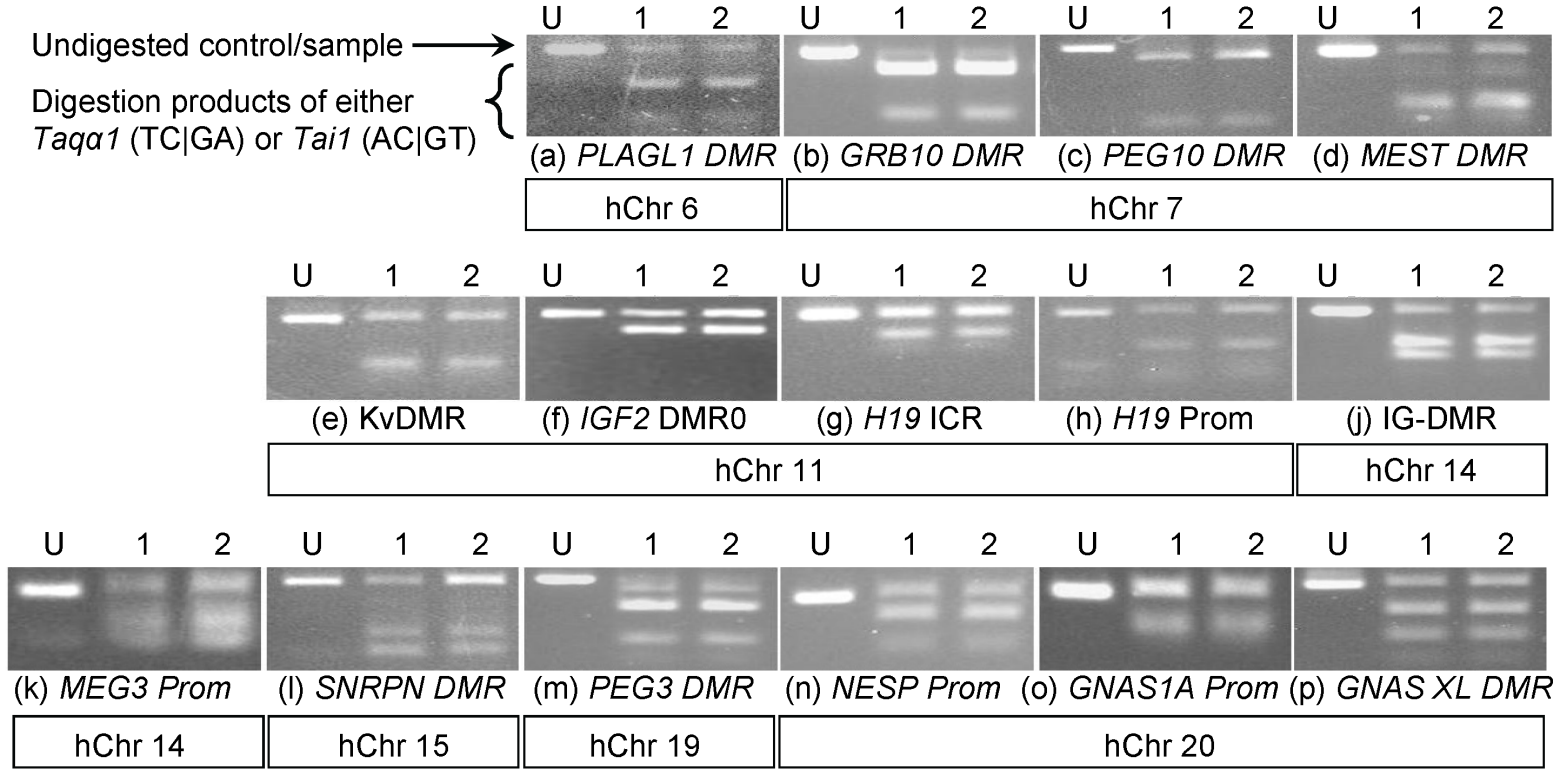
Analysis of differential methylation at imprinted loci using combined bisulphite and restriction analysis (CoBRA). Imprinting control regions at each locus were analysed in two adult PBL samples (PBLC (1) and PBLD (2)) and the presence of a DMR verified by the presence of both undigested and digested amplicons (restriction enzymes used in each case are given in Table S4). Undigested products are representative of unmethylated DNA alleles, where the restriction site is lost through C conversion to T. Digested products indicate the presence of an unmodified CpG dinucleotide in the recognition site, representative of methylated DNA. All differentially methylated regions analysed were maintained, crucially including those ICRs for genes which in our hands were expressed biallelically (b, d, e and o). The DMRs analysed represent both those which are germline (a - e, g, j, l - m, o - p) and somatic (f, h, k and n) in origin, and those methylated on the maternal (a - f, l, m, o and p) and paternal (g, h, j, k and n) alleles.

Monoallelic expression of imprinted genes is controlled by differential methylation at imprinting control regions, (ICRs). We therefore investigated a number of the differentially methylated regions (DMRs) known to be associated with the imprinted genes analysed in this study. Each of the 15 chosen DMRs were analysed by combined bisulphite and restriction analysis (CoBRA) in two individuals and were all found to be maintained (Fig. 3).

**Figure 4:**
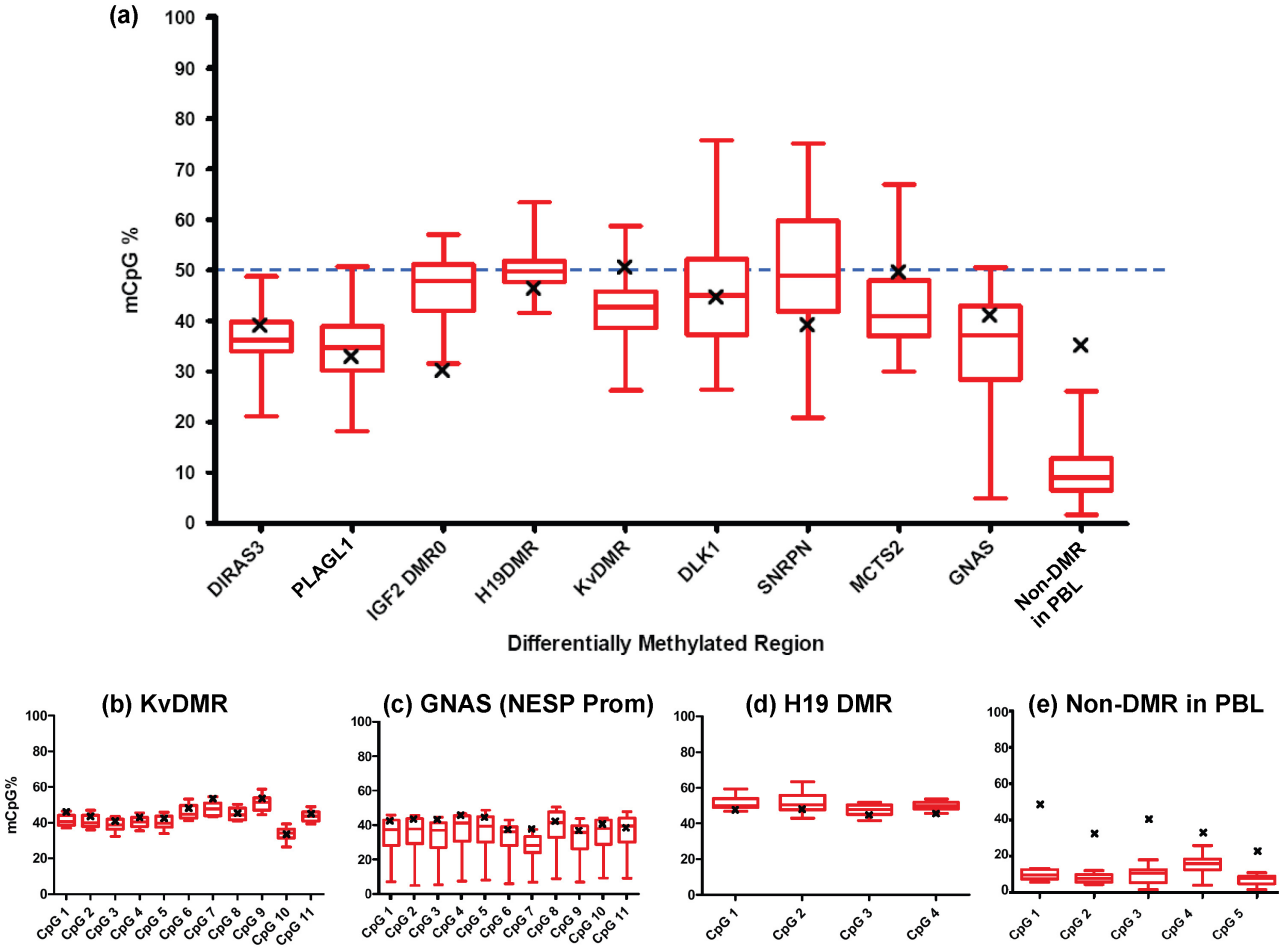
Quantification of DNA methylation at differentially methylated regions (DMRs). (a) Methylation was quantified in the peripheral blood of 12 individuals using a standard pyrosequencing assay for 9 different DMRs and one non-DMR, a region upstream of the *GRB10* gene (18) Average methylation across the DMR regions for all CpGs was calculated. Methylation levels of DMRs in PBL samples shown in red, (n = 12) and brain as black crosses (n = 1). Box plots represent medians and interquartile ranges and max and min values. Data for individual CpGs across the region are illustrated for (b) KvDMR, (c) GNAS NESP Promoter (d) H19 DMR and (e) the non-DMR in PBL. Methylation levels consistent with differential methylation were reported for all nine DMRs examined. In (b), the KvDMR and (c) the NESP promoter, several expressed transcripts in these regions were biallelic. As would be expected, all transcripts associated with the H19 DMR (d) i.e. *IGF2* and *H19* were expressed monoallelically. The non-DMR in PBL (e) was hypomethylated in this tissue and exhibited a tissue-specific methylation increase in brain. CoBRA and pyrosequencing primers were different but colocalised: amplicons were within 50 bp of each other for *SNRPN*; within 1 kb for *MEG3* prom, 500 bp for *KvDMR*, 100 bp for *PLAGL1* 100 bp, overlapped for *NESP* prom, 100 bp for *H19* ICR and 50 bp for *IGF2* DMR0. Each assay included between three and 11 CpGs and remaining individual data for each CpG in all regions are shown in Fig. S4. Pyro Q-CpG Software (Biotage, Uppsala, Sweden) was used to calculate the ratio of converted C's (T's) to unconverted C's at each CpG, this is expressed this as a percentage methylation.

To quantify DMR methylation we also analysed nine DMRs, and one non-DMR, by pyrosequencing in 12 PBL samples. The 12 individuals analysed included those used for quantification of gene expression (PBLA and B) and demonstrated that neither were outliers, shown in Fig. S3. Threshold levels of methylation in these samples were set as described previously (20). This allowed analysis of between three and 11 CpGs per region, where CoBRA permits only one or two. Average methylation across the region for the 12 samples was calculated for each DMR and is displayed in Fig. 4. Individual data for each CpG dinucleotide are shown for KvDMR (b), NESP promoter (c), H19 DMD (d) and the non-DMR (e) in Fig. 4, and the remaining DMRs in Fig. S4. In PBL DNA, each of the DMRs analysed revealed intermediate levels of methylation, ranging between 35 and 50%, which is consistent with methylation levels if only one parental allele is methylated. Methylation at a control region which is not a DMR in blood, and is hypomethylated, is shown with median methylation levels of 10%. An adult brain DNA sample was analysed as a reference tissue sample and methylation levels fell within the interquartile ranges of the methylation detected in PBL, with the exception of *IGF2* DMR0 and KvDMR. The latter DMR in brain had higher methylation levels than blood, but these were still within normal levels as expected for an imprinted gene. Similarly, *IGF2* DMR0 in brain was less methylated compared to PBL but still within the normal threshold levels (Fig. 4). These results together demonstrate the presence of the methylation mark as would be expected for imprinted monoallelic expression. However, biallelic expression is observed for two of these regions in PBL.

### In silico analysis of a gene expression histone signature

To investigate the possibility that histone modifications could be responsible for the regulation of expression at the promoters of imprinted genes with maintained DMRs, we consulted the online human ChIP database to assess the levels of histone methylation and acetylation (http://dir.nhlbi.nih.gov/papers/lmi/epigenomes/hgtcell.html). For almost all of the imprinted genes that we found to be expressed in PBL, a histone signature comprising 17 modifications was present. For genes where we could not detect expression, the signature was partially or completely absent (Fig. S5). Of the imprinted genes analysed, two, *MEST* and *PLAGL1,* are known to have alternatively expressed, biallelic promoters that are active in PBL only. Expression in all other tissues is imprinted and originates from the respective DMR [22], [23]. The histone modification signatures were most pronounced at the PBL-specific transcriptional start sites of *MEST* and *PLAGL1*. These PBL-specific promoters were associated with the full complement of modifications associated with high expression, whereas the DMR associated promoters were not.

## Discussion

In this study we investigate the use of adult human blood as a tissue to detect expression and imprinting status of a number of known imprinted genes. Of the 36 tested only six were detectably imprinted, with the others not proving to be reliable for LOI studies. Principally this was due to a very low level or complete lack of expression, while others were either biallelic or variably expressed in different individuals, despite maintaining differential methylation at respective imprinting control regions. These biallelic expression data were largely unexpected given the monoallelic status of these genes in the human fetus [17], [24]. Previous findings describing extremely low but monoallelic *IGF2* expression and robust monoallelic expression of *SNRPN* in PBL were supported [25], [26].

*In vivo* loss of ICR differential methylation in mice results in a loss of imprinting [27]. In humans, hypo- or hypermethylation of individual loci results in either a loss of, or biallelic, gene expression [28], [29]. However, in adult PBL, we show that a lack of imprinting does not correlate with a loss of differential ICR methylation. Discordant methylation and expected imprinted expression at the *IGF2-H19* locus has been reported in many cancer studies [30]–[32]. Our analysis contained both germline DMRs, such as the *H19* ICR and somatic DMRs which acquire methylation after fertilisation, such as *IGF2* DMR0. Somatic DMRs are more variable in their methylation levels and are also likely to be tissue specific. For example, it has been shown that hypomethylation of DMR0 increases with age and does not result in loss of imprinting of *IGF2* in normal individuals [26]. Methylation of the somatic DMR0 is thought to be controlled by, and indicative of, the methylation state of the *H19* ICR [33].

These observations are suggestive of an uncoupling of methylation and imprinted expression, indicating that an epigenetic mechanism other than DNA methylation may be involved. This has been observed previously in stem cells [34] and in normal tissues, for example *Igf2/IGF2* and *H19* are biallelic in certain regions of the brain despite maintenance of the H19 DMD [35], [36] and also *Kcnq1* expression in mice becomes biallelic after birth despite maintenance of the KvDMR [37]. Recently, a histone signature comprising 17 modifications has been shown to mark the transcriptional start sites of highly expressed genes in T lymphocytes [38]. This histone modification signature correlated almost universally with whether the gene was expressed in PBL or not. Of particular interest are *MEST* and *PLAGL1,* which have alternatively expressed, biallelic promoters that are active in PBL only, whereas expression in all other tissues is imprinted and originates from the respective DMR [22], [23]. This is strongly associated with histone allocation since the PBL-specific transcriptional start sites of *MEST* and *PLAGL1* are associated with the full complement of modifications associated with high expression, whereas they are absent at the DMR associated promoters of these two genes.

The *KCNQ1OT1* transcript is paternally expressed, and biallelic expression of this transcript is caused by a loss of maternal methylation at the KvDMR, as occurs in BWS. This usually leads to silencing of the other imprinted genes in this domain (*SLC22A18, PHLDA2, CDKN1C,* and *KCNQ1*) [39]. We find *KCNQ1OT1* to be universally biallelic amongst PBL samples, however, surprisingly neither *SLC22A18, CDKN1C* nor *KCNQ1* were silenced, and *SLC22A18* and *KCNQ1* were found to also be biallelic. In addition, methylation at the KvDMR was still within normal ranges for a DMR. Analysis of *KCNQ1OT1* and *SLC22A18* allelic expression in the separated T-lymphocyte, B-lymphocyte and myeloid cell sub-populations within PBL found the same biallelic expression pattern in each cell type. Recently the *KCNQ1OT1* ncRNA is was shown to be dispensable for silencing at this locus, specifically in the mouse embryo, dependent only on DNA methylation at the KvDMR, and possibly subsequent CTCF binding [40]. We suggest that this mechanism also occurs in human adult PBL, explaining the continued expression of *KCNQ1* and *CDKN1C* despite biallelic *KCNQ1OT1*.

In a highly proliferative and circulatory cell type such as leukocytes it may be favourable to down-regulate imprinted gene expression, as we observe, either through alternative promoter usage or another epigenetic process i.e. enrichment of repressive histone modifications. The disruption of imprinted gene DMRs has been linked with diseases such as cancer and syndromic imprinting disorders [6], [41]. Our results do not contradict this, as we find no changes in DMR methylation in the PBL of healthy people when compared to another tissue. Subsequently, changes in methylation at multiple imprinted loci in leukocyte DNA, as observed in some cases of BWS and SRS, remain useful for diagnosis of imprinting disorders [42].

It has been suggested that biallelic expression of imprinted genes such as *IGF2* may be a useful predictive marker for risk of colorectal cancer development, even in other, unaffected tissues such as blood [6]. The monoallelic status in normal adult blood of six of the imprinted genes we analysed potentiates their use as a marker, however, of these only *IGF2* has been linked to tumorigenesis. The remaining 30 imprinted genes were either biallelic or below detectable levels in adult peripheral blood leukocytes. Here we demonstrate that many imprinted genes are not highly expressed in blood, and those that are expressed are mostly biallelic. Therefore, a lack of imprinting in adult human blood is both common, normal and in most cases unlikely to provide a useful marker for disease.

### URLs

Imprinted genes: http://igc.otago.ac.nz/

Histone Methylation: http://dir.nhlbi.nih.gov/papers/lmi/epigenomes/hgtcell.html

## Supporting Information

Figure S1 Comparison of imprinted gene (in alphabetical order) expression in fetal tissues, adult PBL and bone marrow. The expression of genes in PBL was quantitatively compared to that in the following tissues: (in order of graph, from left to right) adult bone marrow (very pale grey), fetal brain (pale grey), fetal liver fetal (grey), fetal placenta (dark grey) and adult peripheral blood leukocyte samples A and B (PBLA/B in red). Fetal samples were a mix from two fetuses (Moore fetal tissue cohort) and the adult bone marrow was from a mix of six individuals who had died suddenly (Clontech, CA). The graphs are plotted as in Figure 1, with y = log2-deltaCt. This calculation makes the expression level of GAPDH equal to 1.
(0.06 MB PDF)

Figure S2 Strand specific RT-PCR of KCNQ1OT1 cDNA was created as before except random primers were not included and reverse transcriptase was primed using B-ACTIN and KCNQ1OT1 reverse primers. 2 ug RNA was converted to cDNA using MMLV reverse transcriptase and RT- samples created in parallel with MMLV RT omitted. B-ACTIN RT-PCR for 30 cycles confirmed successful cDNA synthesis without DNA contamination. KCNQ1OT1 RTPCR was carried out for samples informative for the rs231357 A/T polymorphism in KCNQ1OT1 (PBL6, PBL7 and PBL13) using 2 ul of RT+ and RT- for each sample. RT-PCR was carried out for 40 cycles and the amplicons sequenced in the forwards direction. In each case, KCNQ1OT1 was found to be biallelically expressed.
(0.06 MB PDF)

Figure S3 Quantitative measurement of methylation at each DMRs in the two individuals for which expression levels were analysed The two individuals for which we analysed expression of imprinted genes were included in the 12 individuals for which pyrosequencing methylation analysis were carried out on. The max and min bars in Fig. 4 and S4 demonstrate the presence of outliers in the sample, so to check whether these two samples were those outliers - and thus not representative for expression analysis, we created box plots including these samples only (PBLA in Blue, PBLB in Red). Both samples follwed the pattern of the analysis for the pool of 12, there were no large variations between the two samples. There was insufficient DNA in PBL2 to allow analysis of the PLAGL1 DMR so no data point is present for this sample.
(0.27 MB PDF)

Figure S4 Quantitative measurement of methylation at individual CpGs of analysed DMRs A: DIRAS3. B: PLAGL1. C: IGF2 DMR0. D: H19 ICR. E: KvDMR. F: GTL2 Promoter. G: SNRPN. H: MCTS2. I: NESP Promoter J: Non DMR. To quantify DMR methylation we analysed 10 DMRs by pyrosequencing in 12 PBL samples, and compared them to fetal brain. Pyrosequencing allowed analysis of between three and 11 CpGs per region, with methylation levels shown as individual box plots for the PBL samples, and a black cross for the fetal brain sample. Average methylation between the 12 PBL samples is shown as a box plot. Each of the DMRs analysed revealed the expected methylation level for a DMR in PBL and in brain. The non DMR region in (J) was hypomethyated in PBL compared to brain.
(0.09 MB PDF)

Figure S5 Histone modification expression signatures Using the Human March 2006 hg18, assembly (NCBI Build 36.1), high-resolution maps for the genome-wide distribution have been generated (Barski et al., 2007; Wang et al., 2008). Adding each of 17 histone modification tracks found to be associated with expression to the UCSC browser we were able to show that in T-lymphocytes, for genes we find to be expressed in PBL, almost all of the gene promoters are associated with the signature (http://dir.nhlbi.nih.gov/papers/lmi/epigenomes/hgtcell.html),. The signature was not present at promoters of genes which were not expressed. The examples of (i) SNRPN; an expressed gene, and (ii) the two MEST isoforms are shown. Each MEST isoform originates from a separate promoter, one of which is active in blood, the other not.
(0.03 MB PDF)

Table S1 Complete list of QRT-PCR, non quantitative PCR and allelic expression analysis data for all imprinted genes analysed. Prom, promoter; MAT/PAT, maternally/paternally methylated DMR; G/S, DMR established in the germline/post fertilisation; Mat/Pat, maternal/paternal allele expressed; BT, below QPCR detection, i.e. linear amplification was not reached by Threshold, equivalent to a 2-dCt of less than 1x10-12; U, below non-quantitative RT-PCR detection up to 45 cycles; M/B, mono/biallelic; For QPCR: Pl, Placenta; Li, Liver; Br, brain; BM, bone marrow; ND, not done, ‘highest’ indicates that expression relative to the other tissues was highest in PBL. dCtB-dCtA, difference between the dCts of PBLA and PBLB. 2-dCtPBL A/B (3 s.f.), values are shown to 3 significant figures. The fractions in the ‘Allelic Expression’ column indicate the number of biallelic or monoallelic samples there were of the total that were heterozygous (i.e. informative) for each SNP. The number of informative samples represents the total found following genotyping of 50 individuals. *Methylated allele of DMR identified in previously published work, references as shown. ** Parent-of-origin of expression established in previously published work from other laboratories - the various references for imprinted expression and parent-of origin for each gene may be located at http://igc.otago.ac.nz/table.html.
(0.16 MB DOC)

Table S2 Quantitative RT-PCR primers for analysis of gene expression levels. PCR carried out using cDNA template, i.e. post reverse transcription reaction (RT-PCR). Gene transcripts, primers and amplicon size are shown. Each reaction was carried out with a Tm of 60°C for 40 cycles. DM  =  primers designed by Dave Monk, JF  =  primers designed by Jennifer Frost.
(0.09 MB DOC)

Table S3 Allele specific assays for gene expression - detailing SNPs, transcripts, primers and amplicon size. Genotyping, PCR using genomic DNA template; RT, PCR using cDNA template, i.e. post reverse transcription reaction; D, direction; Tm, annealing temperature; JF, primers designed by Jennifer Frost.
(0.14 MB DOC)

Table S4 Bisulphite PCR primers for analysis of differential methylation at imprinted differentially methylated regions. Amplicons were subjected to Combined Bisulphite and Restriction (CoBRA) assays and bisulphite sequencing. Primers are specific for bisulphite converted DNA. Where CpG dinucleotides appear in the primer, 1:1 primer mixes are prepared with either a C or a T base in the forwards direction (YG) and a G or an A base in the reverse (CR). Bisulphite PCR was carried out using Tm of 53 °C, except for KvDMR where the Tm was 50 °C, and all for 45 Cycles. The size/enzyme column indicates the restriction endonuclease used in the CoBRA assays. DM  =  primers designed by Dave Monk, JF  =  primers designed by Jennifer Frost.
(0.09 MB DOC)
