# Supplementary material for: Evaluation of Allelic Expression of Imprinted Genes in Adult Human Blood
Source: PLoS One. 2010 Oct 21;5(10):e13556. doi: 10.1371/journal.pone.0013556 (PMC2958851; doi:10.1371/journal.pone.0013556)
Supplement: Figure S1 — Comparison of imprinted gene (in alphabetical order) expression in fetal tissues, adult PBL and bone marrow. The expression of genes in PBL was quantitatively compared to that in the following tissues: (in order of graph, from left to right) adult bone marrow (very pale grey), fetal brain (pale grey), fetal liver fetal (grey), fetal placenta (dark grey) and adult peripheral blood leukocyte samples A and B (PBLA/B in red). Fetal samples were a mix from two fetuses (Moore fetal tissue cohort) and the adult bone marrow was from a mix of six individuals who had died suddenly (Clontech, CA). The graphs are plotted as in Figure 1, with y = log2-deltaCt. This calculation makes the expression level of GAPDH equal to 1. (0.06 MB PDF) [file pone.0013556.s001.pdf]

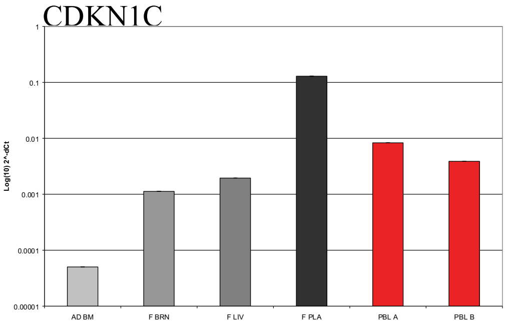
CDKN1C
Log(10) 2^-dCt
1
0.1
0.01
0.001
0.0001
0.00001
AD BM
F BRN
F LIV
F PLA
PBL A
PBL B
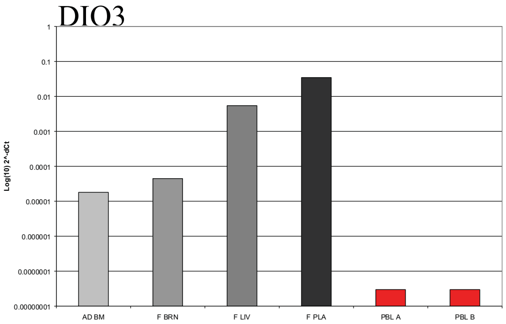
DIO3
Log(10) 2^-dCt
1
0.1
0.01
0.001
0.0001
0.00001
0.000001
0.0000001
0.00000001
AD BM
F BRN
F LIV
F PLA
PBL A
PBL B
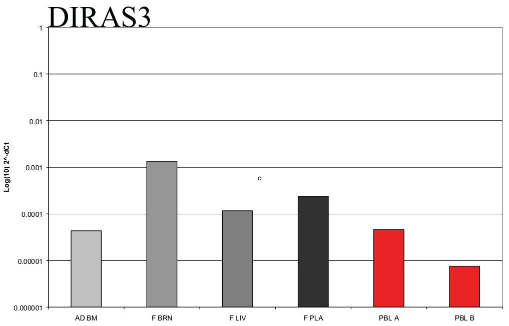
DIRAS3
Log(10) 2^-dCt
1
0.1
0.01
0.001
0.0001
0.00001
0.000001
AD BM
F BRN
F LIV
F PLA
PBL A
PBL B
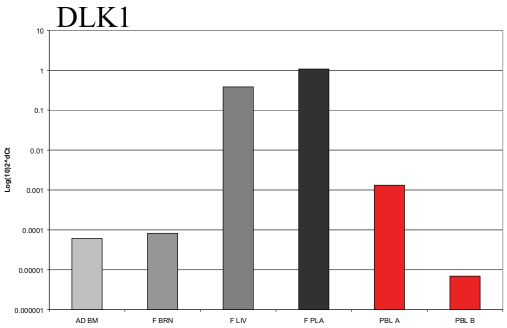
DLK1
Log(10)2^dCt
10
1
0.1
0.01
0.001
0.0001
0.00001
0.000001
AD BM
F BRN
F LIV
F PLA
PBL A
PBL B
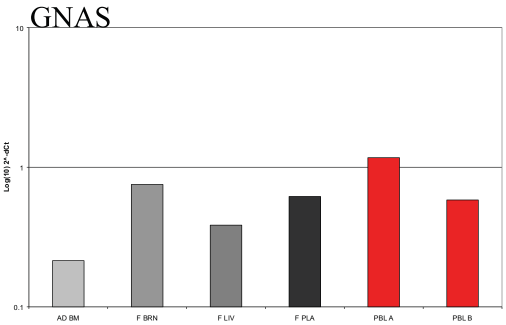
GNAS
Log(10) 2^-dCt
10
1
0.1
AD BM
F BRN
F LIV
F PLA
PBL A
PBL B
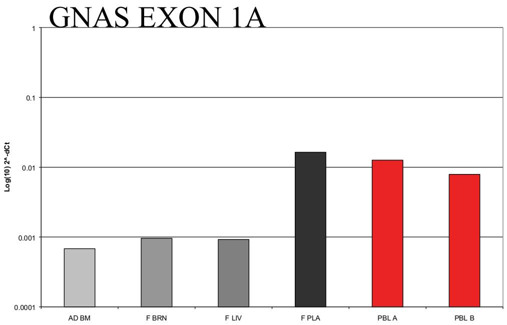
GNAS EXON 1A
Log(10) 2^-dCt
1
0.1
0.01
0.001
0.0001
AD BM
F BRN
F LIV
F PLA
PBL A
PBL B
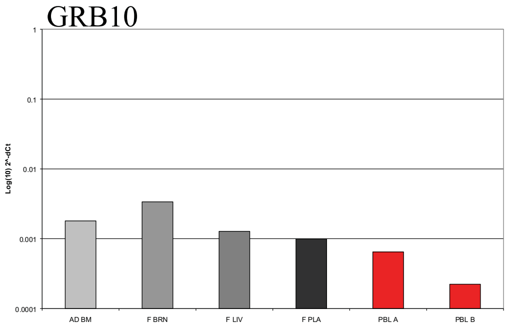
GRB10
Log(10) 2^-dCt
1
0.1
0.01
0.001
0.0001
AD BM
F BRN
F LIV
F PLA
PBL A
PBL B
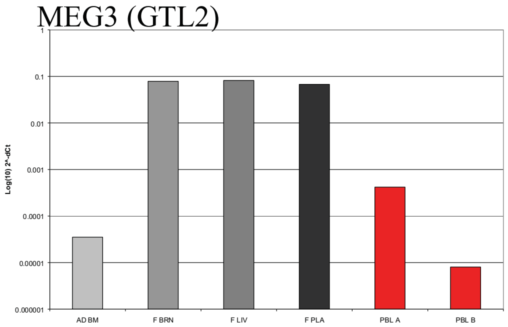
MEG3 (GTL2)
Log(10) 2^-dCt
1
0.1
0.01
0.001
0.0001
0.00001
0.000001
AD BM
F BRN
F LIV
F PLA
PBL A
PBL B
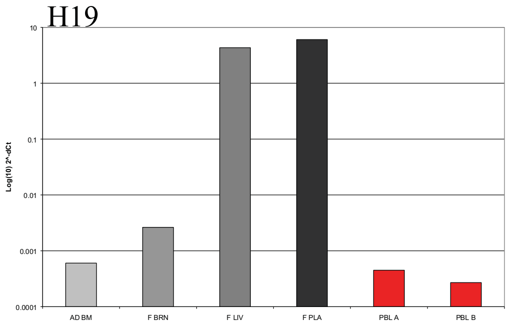
H19
Log(10) 2^-dCt
10
1
0.1
0.01
0.001
0.0001
AD BM
F BRN
F LIV
F PLA
PBL A
PBL B
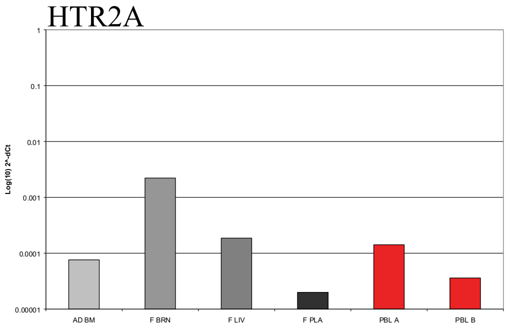
HTR2A
Log(10) 2^-dCt
1
0.1
0.01
0.001
0.0001
0.00001
AD BM
F BRN
F LIV
F PLA
PBL A
PBL B

IGF2

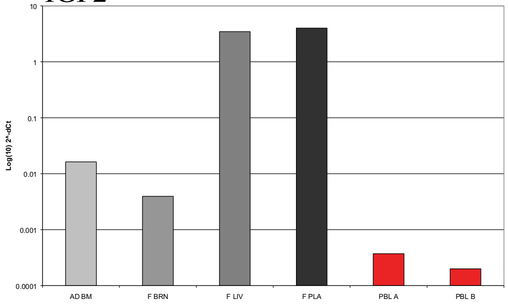

IGF2R

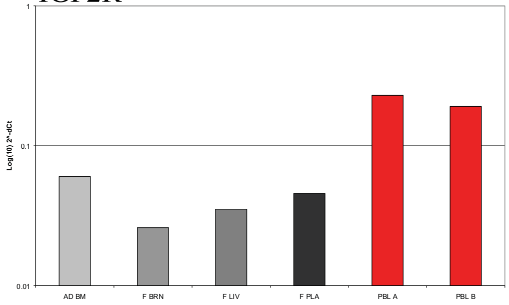

IPW

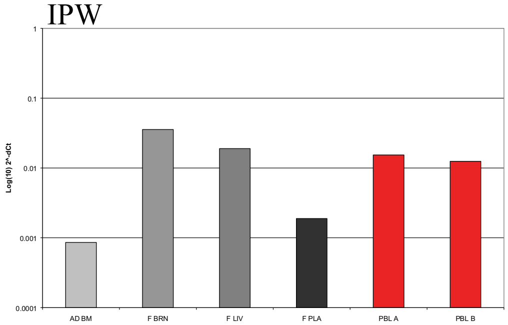

KCNQ1

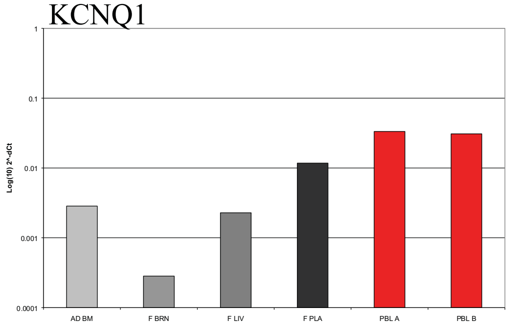

KCNQ1OT1

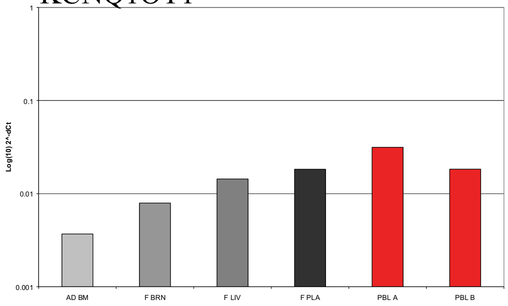

MCTS2

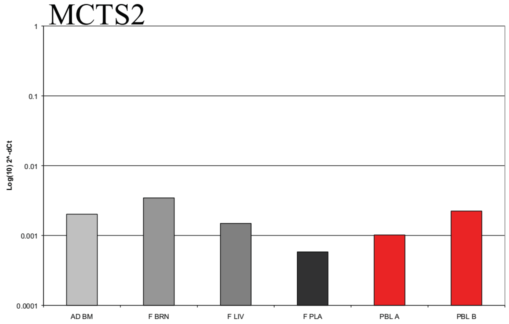

MEST Isoform 1

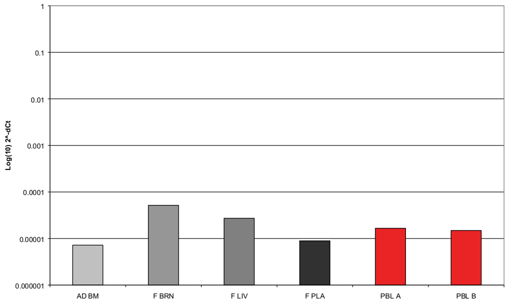

MEST Isoform 2

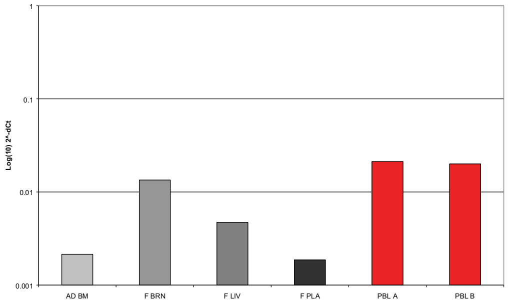

MESTIT1

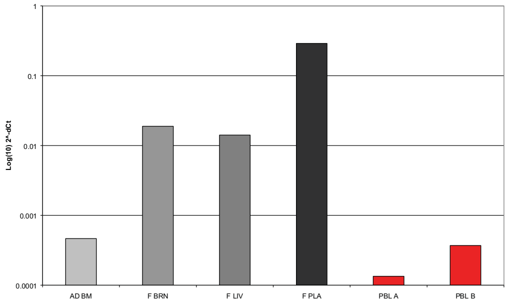

NAP1L5

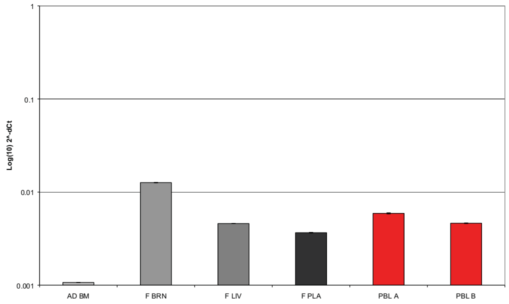

NDN

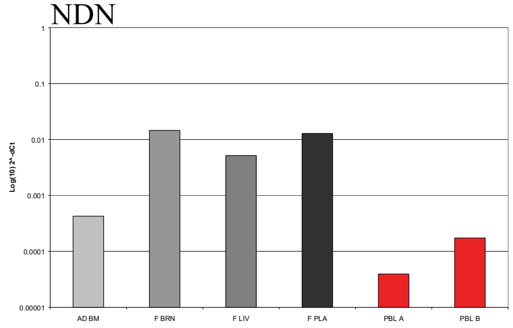

NESP

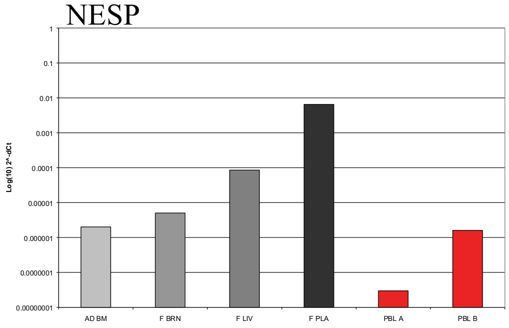

NNAT

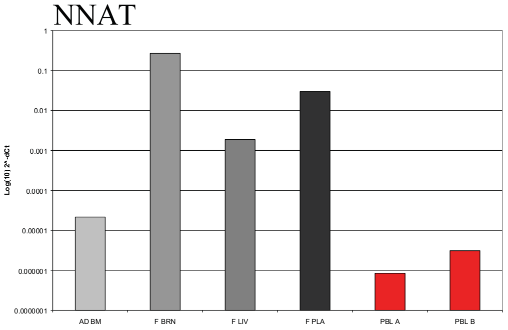

PEG3

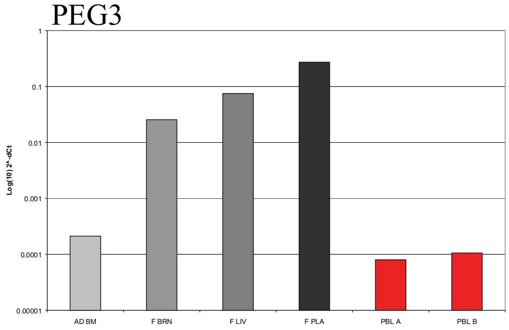

PEG10

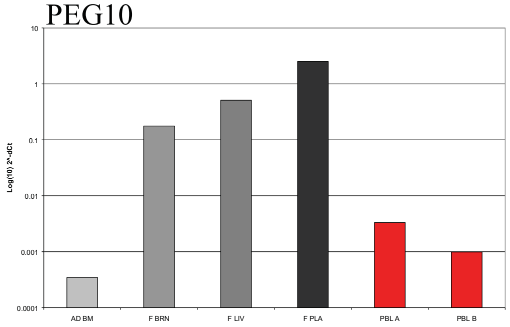

PHLDA2

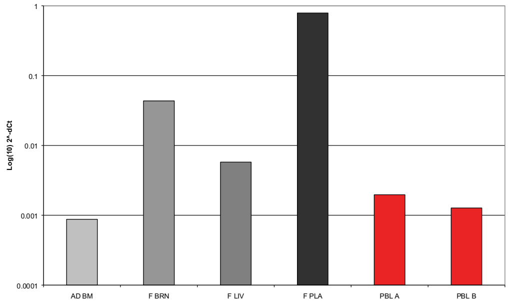

SGCE

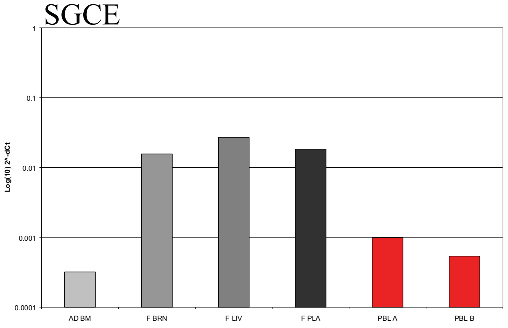

SLC22A18

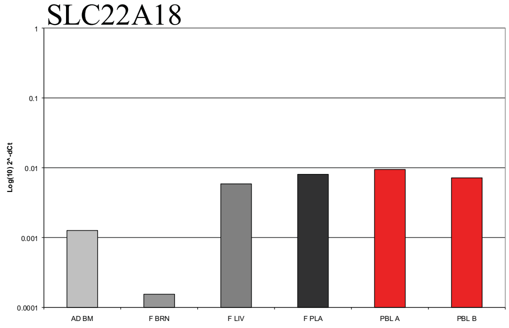

SNRPN

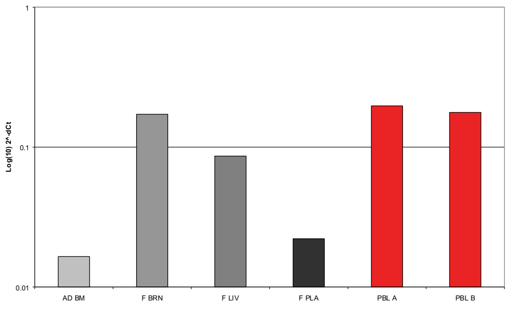

TP73

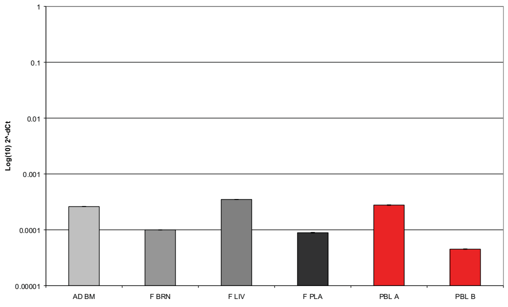

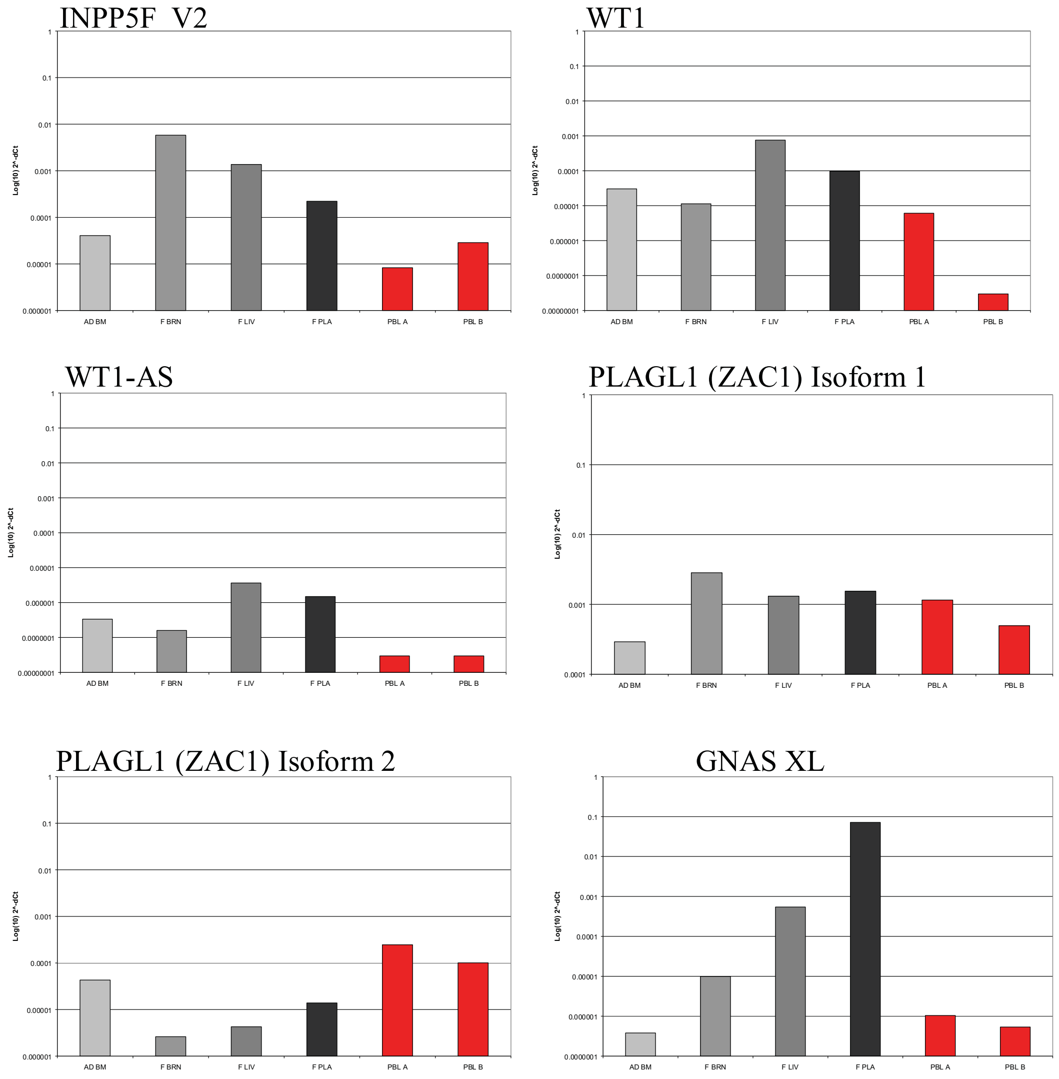

S1. Comparison of imprinted gene (in alphabetical order) expression in fetal tissues, adult PBL and bone marrow. The expression of genes in PBL was quantitatively compared to that in the following tissues: (in order of graph, from left to right) adult bone marrow (very pale grey), fetal brain (pale grey), fetal liver fetal (grey), fetal placenta (dark grey) and adult peripheral blood leukocyte samples A and B (PBLA/B in red). Fetal samples were a mix from two fetuses (Moore fetal tissue cohort) and the adult bone marrow was from a mix of six individuals who had died suddenly (Clontech, CA). The graphs are plotted as in Figure 1a, with $y=\log 2^{-\Delta Ct}$. This calculation makes the expression level of *GAPDH* equal to 1.
