# Supplementary material for: Evaluation of Allelic Expression of Imprinted Genes in Adult Human Blood
Source: PLoS One. 2010 Oct 21;5(10):e13556. doi: 10.1371/journal.pone.0013556 (PMC2958851; doi:10.1371/journal.pone.0013556)
Supplement: Figure S2 — Strand specific RT-PCR of KCNQ1OT1 cDNA was created as before except random primers were not included and reverse transcriptase was primed using B-ACTIN and KCNQ1OT1 reverse primers. 2 ug RNA was converted to cDNA using MMLV reverse transcriptase and RT- samples created in parallel with MMLV RT omitted. B-ACTIN RT-PCR for 30 cycles confirmed successful cDNA synthesis without DNA contamination. KCNQ1OT1 RTPCR was carried out for samples informative for the rs231357 A/T polymorphism in KCNQ1OT1 (PBL6, PBL7 and PBL13) using 2 ul of RT+ and RT- for each sample. RT-PCR was carried out for 40 cycles and the amplicons sequenced in the forwards direction. In each case, KCNQ1OT1 was found to be biallelically expressed. (0.06 MB PDF) [file pone.0013556.s002.pdf]

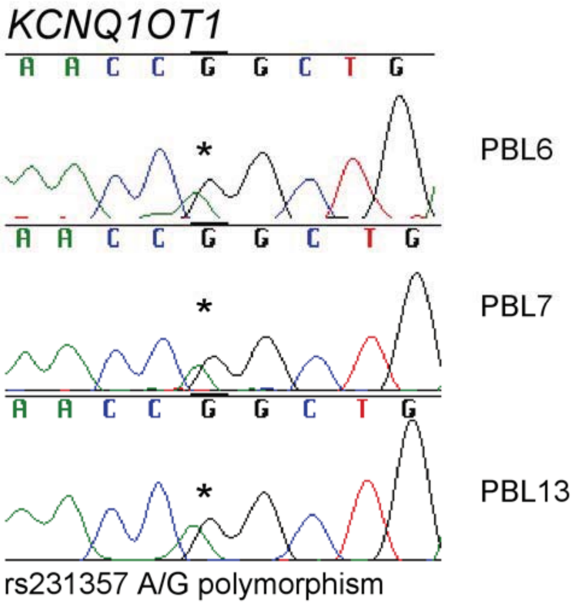

S2. Strand specific RT-PCR of *KCNQ1OT1*

cDNA was created as before except random primers were not included and reverse transcriptase was primed using *B-ACTIN* and *KCNQ1OT1* reverse primers. 2 ug RNA was converted to cDNA using MMLV reverse transcriptase and RT- samples created in parallel with MMLV RT omitted. *B-ACTIN* RT-PCR for 30 cycles confirmed successful cDNA synthesis without DNA contamination. *KCNQ1OT1* RT-PCR was carried out for samples informative for the rs231357 A/T polymorphism in *KCNQ1OT1* (PBL6, PBL7 and PBL13) using 2 ul of RT+ and RT- for each sample. RT-PCR was carried out for 40 cycles and the amplicons sequenced in the forwards direction. In each case, KCNQ1OT1 was found to be biallelically expressed.
