# Supplementary material for: Evaluation of Allelic Expression of Imprinted Genes in Adult Human Blood
Source: PLoS One. 2010 Oct 21;5(10):e13556. doi: 10.1371/journal.pone.0013556 (PMC2958851; doi:10.1371/journal.pone.0013556)
Supplement: Figure S3 — Quantitative measurement of methylation at each DMRs in the two individuals for which expression levels were analysed The two individuals for which we analysed expression of imprinted genes were included in the 12 individuals for which pyrosequencing methylation analysis were carried out on. The max and min bars in Fig. 4 and S4 demonstrate the presence of outliers in the sample, so to check whether these two samples were those outliers - and thus not representative for expression analysis, we created box plots including these samples only (PBLA in Blue, PBLB in Red). Both samples follwed the pattern of the analysis for the pool of 12, there were no large variations between the two samples. There was insufficient DNA in PBL2 to allow analysis of the PLAGL1 DMR so no data point is present for this sample. (0.27 MB PDF) [file pone.0013556.s003.pdf]

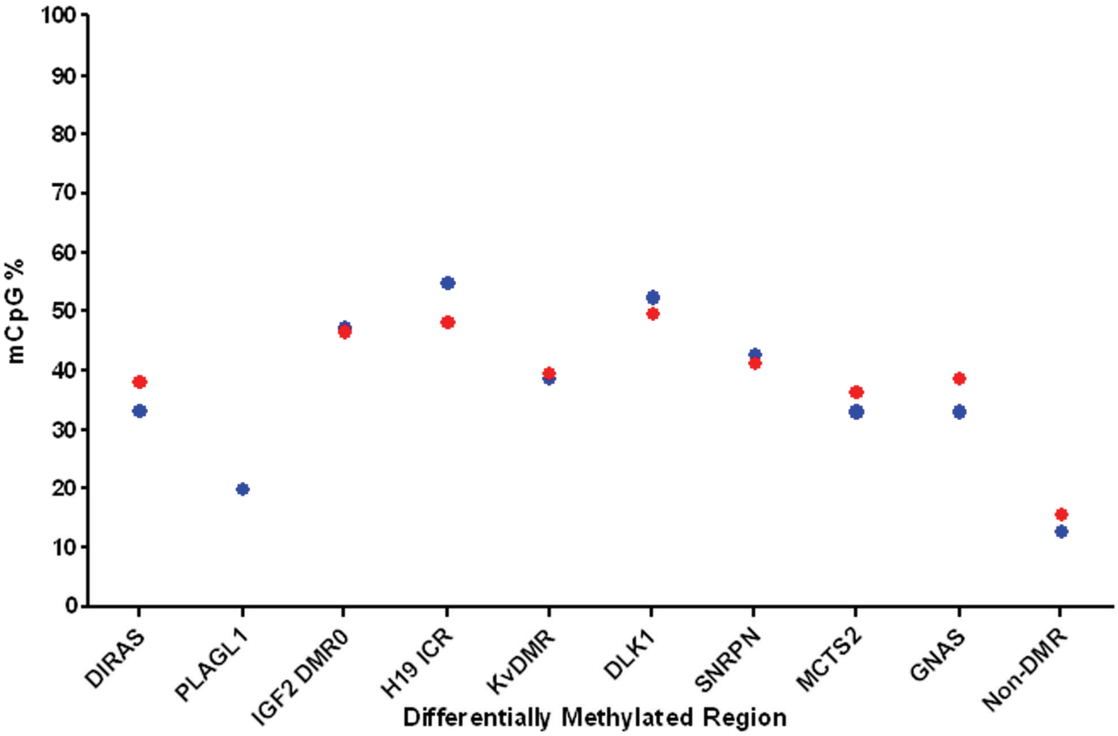

S3. Quantitative measurement of methylation at each DMRs in the two individuals for which expression levels were analysed

The two individuals for which we analysed expression of imprinted genes were included in the 12 individuals for which pyrosequencing methylation analysis were carried out on. The max. and min. bars in Figs 4 and S4 demonstrate the presence of outliers in the sample, so to check whether these two samples were those outliers – and thus not representative for expression analysis, we created box plots including these samples only (PBLA in Blue, PBLB in Red). Both samples follwed the pattern of the analysis for the pool of 12, there were no large variations between the two samples. There was insufficient DNA in PBL2 to allow analysis of the PLAGL1 DMR so no data point is present for this sample.
