# Supplementary material for: Evaluation of Allelic Expression of Imprinted Genes in Adult Human Blood
Source: PLoS One. 2010 Oct 21;5(10):e13556. doi: 10.1371/journal.pone.0013556 (PMC2958851; doi:10.1371/journal.pone.0013556)
Supplement: Figure S4 — Quantitative measurement of methylation at individual CpGs of analysed DMRs A: DIRAS3. B: PLAGL1. C: IGF2 DMR0. D: H19 ICR. E: KvDMR. F: GTL2 Promoter. G: SNRPN. H: MCTS2. I: NESP Promoter J: Non DMR. To quantify DMR methylation we analysed 10 DMRs by pyrosequencing in 12 PBL samples, and compared them to fetal brain. Pyrosequencing allowed analysis of between three and 11 CpGs per region, with methylation levels shown as individual box plots for the PBL samples, and a black cross for the fetal brain sample. Average methylation between the 12 PBL samples is shown as a box plot. Each of the DMRs analysed revealed the expected methylation level for a DMR in PBL and in brain. The non DMR region in (J) was hypomethyated in PBL compared to brain. (0.09 MB PDF) [file pone.0013556.s004.pdf]

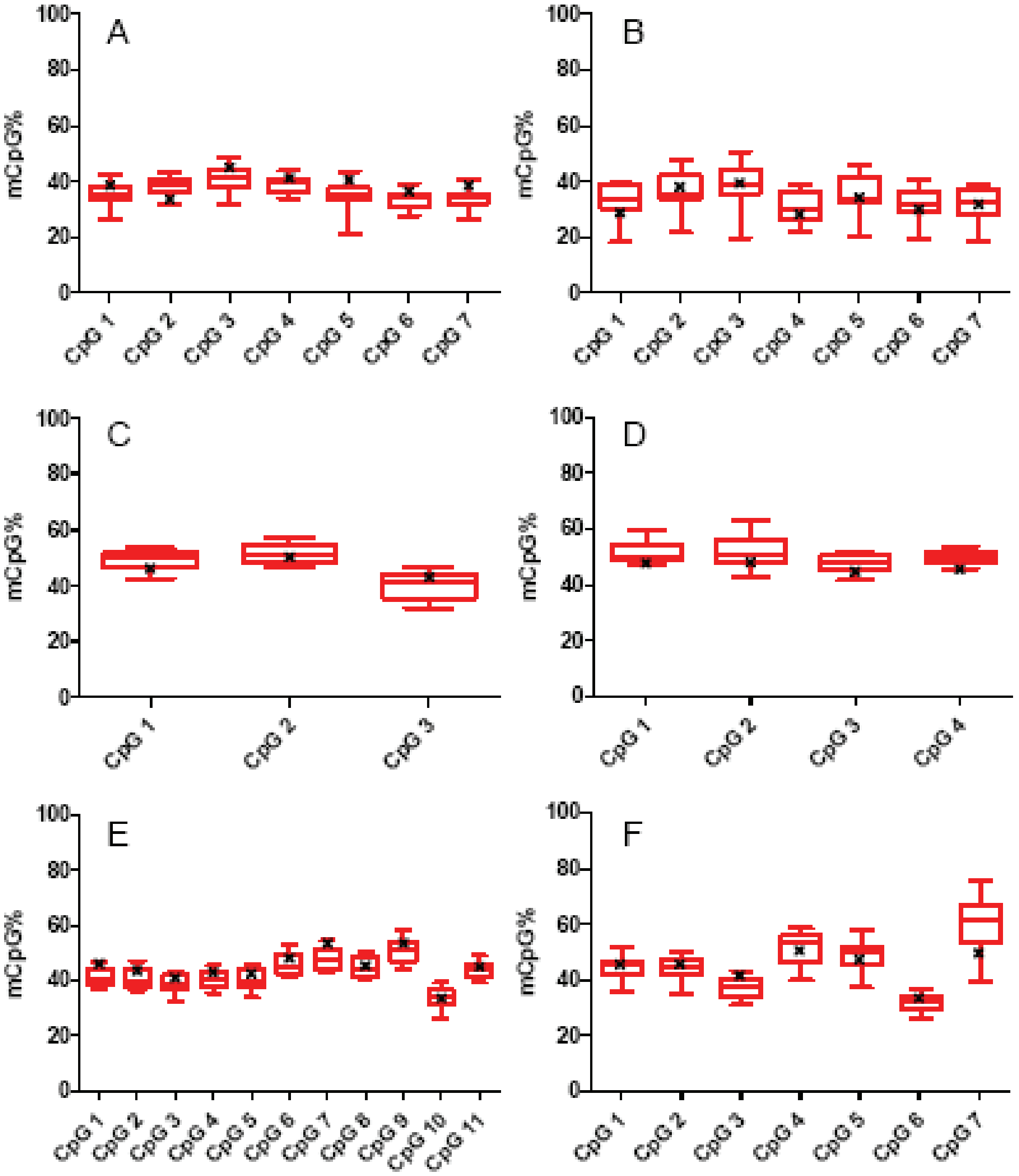
A
B
C
D
E
F
mCpG%
100
80
60
40
20
0
CpG 1
CpG 2
CpG 3
CpG 4
CpG 5
CpG 6
CpG 7
CpG 8
CpG 9
CpG 10
CpG 11

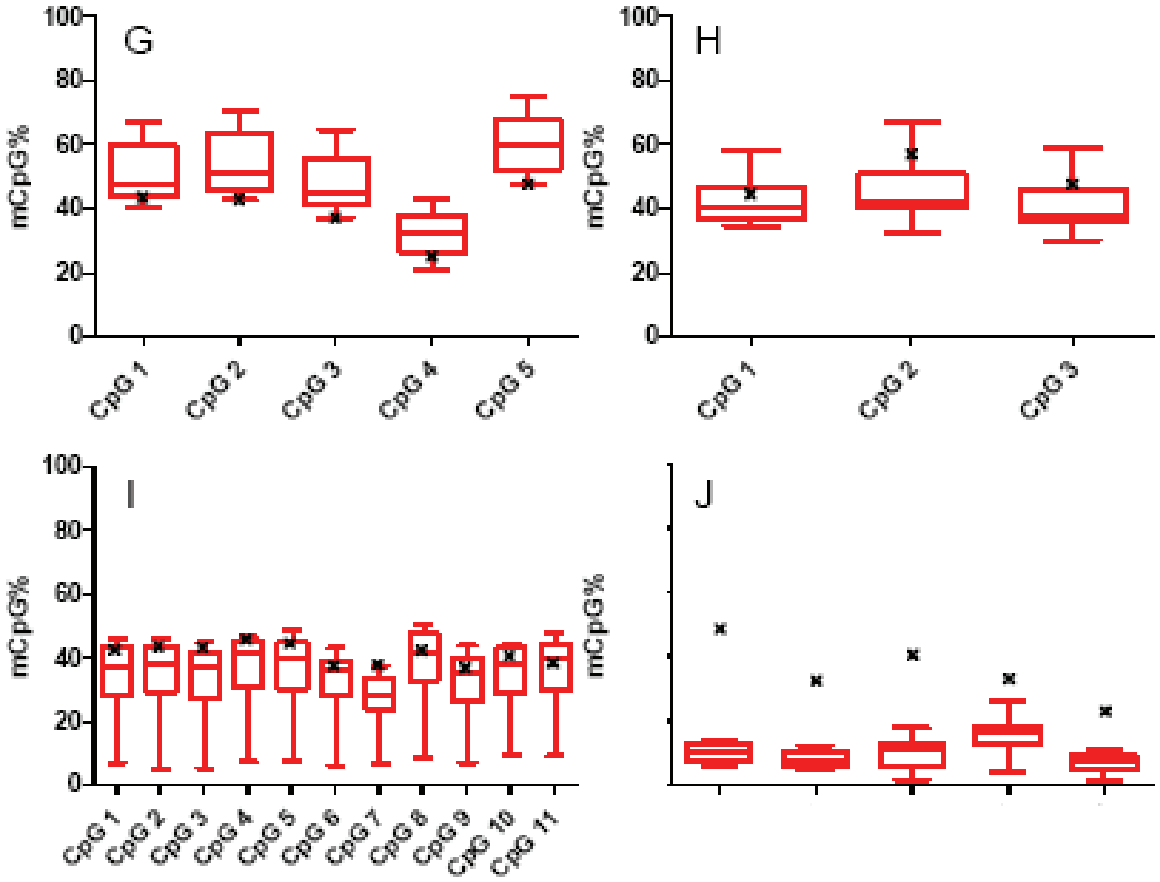

S4. Quantitative measurement of methylation at individual CpGs of analysed DMRs

A: DIRAS3. B: PLAGL1. C: IGF2 DMR0. D: H19 ICR. E: KvDMR. F: MEG3 Promoter. G: SNRPN. H: MCTS2. I: NESP Promoter J: Non DMR. To quantify DMR methylation we analysed 10 DMRs by pyrosequencing in 12 PBL samples, and compared them to fetal brain. Pyrosequencing allowed analysis of between three and 11 CpGs per region, with methylation levels shown as individual box plots for the PBL samples, and a black cross for the fetal brain sample. Average methylation between the 12 PBL samples is shown as a box plot. Each of the DMRs analysed revealed the expected methylation level for a DMR in PBL and in brain. The non DMR region in (J) was hypomethyated in PBL compared to brain.
