# Supplementary material for: Evaluation of Allelic Expression of Imprinted Genes in Adult Human Blood
Source: PLoS One. 2010 Oct 21;5(10):e13556. doi: 10.1371/journal.pone.0013556 (PMC2958851; doi:10.1371/journal.pone.0013556)
Supplement: Figure S5 — Histone modification expression signatures Using the Human March 2006 hg18, assembly (NCBI Build 36.1), high-resolution maps for the genome-wide distribution have been generated (Barski et al., 2007; Wang et al., 2008). Adding each of 17 histone modification tracks found to be associated with expression to the UCSC browser we were able to show that in T-lymphocytes, for genes we find to be expressed in PBL, almost all of the gene promoters are associated with the signature (http://dir.nhlbi.nih.gov/papers/lmi/epigenomes/hgtcell.html),. The signature was not present at promoters of genes which were not expressed. The examples of (i) SNRPN; an expressed gene, and (ii) the two MEST isoforms are shown. Each MEST isoform originates from a separate promoter, one of which is active in blood, the other not. (0.03 MB PDF) [file pone.0013556.s005.pdf]

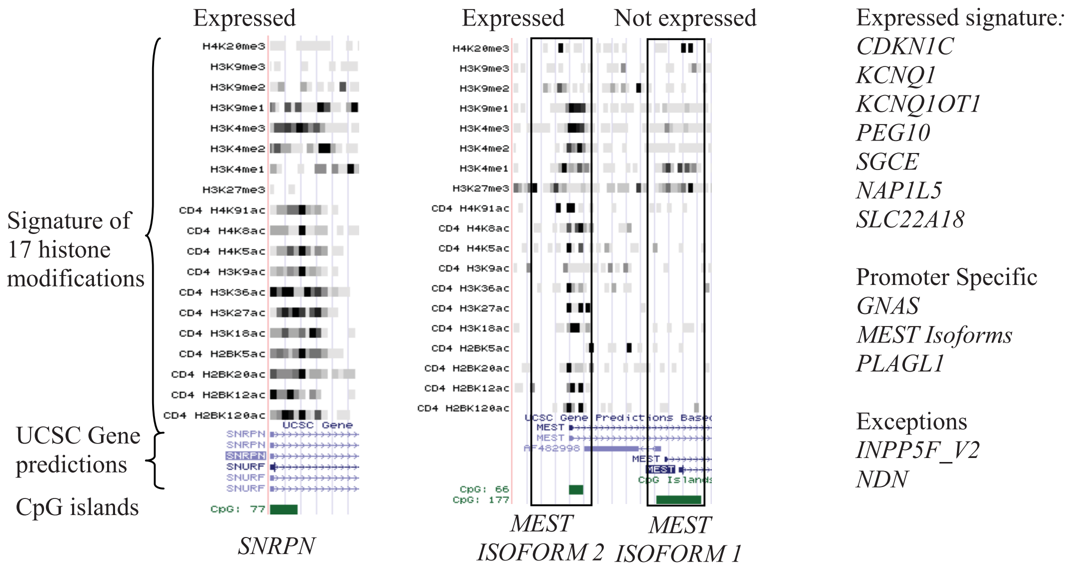

S5. Histone modification expression signatures

Using the Human March 2006 hg18, assembly (NCBI Build 36.1), high-resolution maps for the genome-wide distribution have been generated (Barski et al., 2007; Wang et al., 2008). Adding each of 17 histone modification tracks found to be associated with expression to the UCSC browser we were able to show that in T-lymphocytes, for genes we find to be expressed in PBL, almost all of the gene promoters are associated with the signature (http://dir.nhlbi.nih.gov/papers/lmi/epigenomes/hgtcell.html),. The signature was not present at promoters of genes which were not expressed. The examples of (i) *SNRPN*; an expressed gene, and (ii) the two *MEST* isoforms are shown. Each *MEST* isoform originates from a separate promoter, one of which is active in blood, the other not.
