# Supplementary material for: Evaluation of Allelic Expression of Imprinted Genes in Adult Human Blood
Source: PLoS One. 2010 Oct 21;5(10):e13556. doi: 10.1371/journal.pone.0013556 (PMC2958851; doi:10.1371/journal.pone.0013556)
Supplement: Table S1 — Complete list of QRT-PCR, non quantitative PCR and allelic expression analysis data for all imprinted genes analysed. Prom, promoter; MAT/PAT, maternally/paternally methylated DMR; G/S, DMR established in the germline/post fertilisation; Mat/Pat, maternal/paternal allele expressed; BT, below QPCR detection, i.e. linear amplification was not reached by Threshold, equivalent to a 2-dCt of less than 1x10^-12^; U, below non-quantitative RT-PCR detection up to 45 cycles; M/B, mono/biallelic; For QPCR: Pl, Placenta; Li, Liver; Br, brain; BM, bone marrow; ND, not done, ‘highest’ indicates that expression relative to the other tissues was highest in PBL. dCtB-dCtA, difference between the dCts of PBLA and PBLB. 2-dCtPBL A/B (3 s.f.), values are shown to 3 significant figures. The fractions in the ‘Allelic Expression’ column indicate the number of biallelic or monoallelic samples there were of the total that were heterozygous (i.e. informative) for each SNP. The number of informative samples represents the total found following genotyping of 50 individuals. *Methylated allele of DMR identified in previously published work, references as shown. ** Parent-of-origin of expression established in previously published work from other laboratories - the various references for imprinted expression and parent-of origin for each gene may be located at http://igc.otago.ac.nz/table.html. (0.16 MB DOC) [file pone.0013556.s006.doc]

**Table S1**

| Gene/  cluster | Locus | Transcripts in cluster | UCSC SNP annotation | Methylated DMR* | P of O** | dCt PBL A | dCt PBL B | **dCtB-dCtA** | 2^-dCt^PBL A  (3 s.f.) | 2^-dCt^PBL B  (3 s.f.) | QPCR | RT PCR | Allelic Exp |
| --- | --- | --- | --- | --- | --- | --- | --- | --- | --- | --- | --- | --- | --- |
| *DIRAS3* | 1p31 |  |  | [1] Prom MAT | Pat | 14.411 | 17.017 | 2.61 | 4.59E-05 | 7.54E-06 | BT | U | - |
| *TP73* | 1p36 |  |  | Prom PAT | Mat | 11.814 | 14.436 | 2.62 | 0.000278 | 4.51E-05 | BT | U | - |
| *NAP1L5* | 4q22 |  | rs710834 | [2]Prom MAT^G^ | Pat | 5.555 | 5.645 | 0.09 | 0.0213 | 0.0200 | Highest | 40 | 9/9 B |
| *ZAC1* | 6q24 | Isoform 1 | rs9373409 | [3]Prom MAT^G^ | Pat | 9.759 | 10.977 | 1.22 | 0.00115 | 0.000496 | Pl, Li, Br | U | - |
|  |  | Isoform 2 |  |  | B | 11.988 | 13.277 | 1.29 | 0.000246 | 0.000101 | BT | 45 | 3/3 B |
| *IGF2R* | 6q25 |  | rs614754 |  | Mat | 2.389 | 2.125 | -0.26 | 0.191 | 0.229 | Highest |  | 1/1 B |
| *GRB10* | 7p11 | GRB10 | rs1800504 | **[4]CGI2 MAT^G^** | Pat | 10.593 | 12.13 | 1.54 | 0.000647 | 0.000223 | BT | U | - |
| *PEG10* | 7q21 | *PEG10* | *rs13073* | [5]Prom MAT^G^ | Pat | 8.231 | 9.988 | 1.76 | 0.00333 | 0.000985 | Pl, Li, Br | 40 | 4/4 M |
|  |  | *SGCE* |  |  | Pat | 9.971 | 10.86 | 0.89 | 0.000996 | 0.000538 | BT | U | - |
| *MEST* | 7q32 | *CPA4* | *rs2171492* |  | Mat | ND | ND | ND | ND | ND | - | U | - |
|  |  | *MESTIT1* | *rs12706940* | [6]Prom MAT^G^ | Pat | 15.879 | 16.034 | 0.15 | 1.66E-05 | 1.49E-05 | BT | U | - |
|  |  | *MEST* Isoform 1 | rs1050582 |  | Pat | 12.866 | 11.401 | -1.47 | 0.000134 | 0.000370 | BT | U | - |
|  |  | *MEST* Isoform 2 |  |  | Pat | 7.403 | 7.756 | 0.35 | 0.00591 | 0.00463 | Br | 35 | 8/9 B; 1/9 M |
|  |  | *KLF14* | *agaagcc(a/g)gacgagg* | Sugg DMR^G^PAT | Mat | ND | ND | ND | ND | ND | - | U | - |
| *INPP5F*  *_V2* | 10q26 |  | *rs3188055* | [2]Prom MAT^G^ | Pat | 16.885 | 15.091 | -1.79 | 8.26E-06 | 2.87E-05 | BT | 40 | 8/11 M; 3/11 B |
| *WT1* | 11p13 |  |  | [7]ARR DMR MAT | Pat | 17.324 | U (>40) | U (>40) | 6.09E-06 | <1x10^12^ | BT | U | - |
| *WT1-AS* |  |  |  |  |  | U (>40) | U (>40) | U (>40) | <1x10^-12^ | <1x10^12^ | BT | U | - |
| *KCNQ1* | 11p15 | *PHLDA2* |  | [8]KvDMR^G^MAT | Mat | 8.989 | 9.617 | 0.63 | 0.00197 | 0.00127 | Pl, Li, Br | U | - |
|  |  | *SLC22A18* | *rs1048046/7* |  | Mat | 6.726 | 9.617 | 2.89 | 0.00945 | 0.00127 | Highest | 40 | 5/5 B |
|  |  | *SLC22A1LS* |  |  | Mat | ND | ND | ND | ND | ND | - | U | - |
|  |  | *CDKN1C* |  |  | Mat | 6.91 | 8.011 | 1.10 | 0.00832 | 0.00388 | Pl | U | - |
|  |  | *KCNQ1OT1* | *rs231357/9* |  | Pat | 4.991 | 5.77 | 0.78 | 0.0314 | 0.0183 | Highest/Pl | 40 | 6/6 B |
|  |  | *KCNQ1* | rs1057128 |  | Mat | 4.907 | 5.021 | 0.11 | 0.0333 | 0.0308 | Highest | 38 | 4/4 B |
| *H19/IGF2* | 11p15 | *H19* |  | [9]DMR1 PAT^G^  P0 PAT^S^, DMR2 PAT^S^ | Mat | 11.118 | 11.854 | 0.74 | 0.000450 | 0.000270 | BT | U | - |
|  |  | *IGF2* | *rs680* |  | Pat | 11.391 | 12.29 | 0.90 | 0.000372 | 0.000200 | Pl, Li, Br, BM | 33 | 20/20 M |
| *HTRA2* | 13q14 |  |  |  | Mat | 12.782 | 14.758 | 1.98 | 0.000142 | 3.61E-05 | BT | U | - |
| *DLK1/*  *DIO3* | 14q32 | *GTL2* |  | [10]GTL2 prom PAT^S^  IGDMR^G^PAT | Mat | 11.223 | 16.924 | 5.70 | 0.000418 | 8.04E-06 | BT | U | - |
|  |  | *DLK1* |  |  | Pat | 9.569 | 17.139 | 7.57 | 0.00132 | 6.93E-06 | Pl, Li | U | - |
|  |  | *DIO3* |  |  | Pat | U (>40) | U (>40) | U (>40) | <1x10^-12^ | <1x10^12^ | BT | U | - |
| *SNURF/*  *SNRPN* | 15q11 | *NDN* | *rs2192206* | Prom MAT? | Pat | 14.632 | 12.495 | -2.14 | 3.94E-05 | 0.000173 | BT | 40 | 3/3 M |
|  |  | *SNRPN* | *rs705* | [11]Prom MAT^G^ | Pat | 2.344 | 2.499 | 0.16 | 0.197 | 0.177 | Highest | 40 | 3/3 M |
|  |  | *IPW* | *rs691* |  | Pat | 6.024 | 6.326 | 0.30 | 0.0154 | 0.0125 | Br, Li | 35 | 5/5M |
|  |  | *ATP10C* |  |  | Mat | ND | ND | ND | ND | ND | - | U | - |
| *PEG3* | 19q13 |  |  | [12]Prom MAT^G^ | Pat | 13.611 | 13.207 | -0.40 | 7.99E-05 | 0.000106 | BT | U | - |
| *MCTS2* | 20q11 |  |  | [2]Prom MAT^G^ | Pat | 9.939 | 8.803 | -1.14 | 0.000581 | 0.00102 | BT | U | - |
| *NNAT* | 20q11 |  |  |  | Mat | 20.179 | 18.301 | -1.88 | 8.42E-07 | 3.10E-06 | BT | U | - |
| *GNAS* | 20q13 | *NESP* |  | [13, 14] NESP55 PAT^S^ | Mat | U (>40) | 19.255 | U (>40) | <1x10^-12^ | 1.60E-06 | BT | U | - |
|  |  | *GNAS* | *rs7121* | [15]1A Prom MAT^G^ | Mat | 0.78 | -0.227 | -1.01 | 0.582 | 1.17 | Highest/Pl, Br | 35 | 13/13 B |
|  |  | *Exon 1A* |  | [16]1A Prom MAT^G^ | Pat | 6.306 | 6.984 | 0.68 | 0.0126 | 0.00790 | Pl | 40 | 7/7 M |
|  |  | *GNAS XL* |  | [13, 17] XL Prom MAT^G^ | Pat | 19.871 | 20.833 | 0.96 | 1.04E-06 | 5.35E-07 | BT | U | - |

**Table S1 Complete list of QRT-PCR, non quantitative PCR and allelic expression analysis data for all imprinted genes analysed.**

Prom, promoter; MAT/PAT, maternally/paternally methylated DMR; ^G/S^, DMR established in the germline/post fertilisation; Mat/Pat, maternal/paternal allele expressed; BT, below QPCR detection, i.e. linear amplification was not reached by Threshold, equivalent to a 2^-dCt^ of less than 1x10^-12^; U, below non-quantitative RT-PCR detection up to 45 cycles; M/B, mono/biallelic; For QPCR: Pl, Placenta; Li, Liver; Br, brain; BM, bone marrow; ND, not done, ‘highest’ indicates that expression relative to the other tissues was highest in PBL. dCtB-dCtA, difference between the dCts of PBLA and PBLB. 2^-dCt^PBL A/B (3 s.f.), values are shown to 3 significant figures. The fractions in the ‘Allelic Expression’ column indicate the number of biallelic or monoallelic samples there were of the total that were heterozygous (i.e. informative) for each SNP. The number of informative samples represents the total found following genotyping of 50 individuals. *Methylated allele of DMR identified in previously published work, references as shown. ** Parent-of-origin of expression established in previously published work from other laboratories – the various references for imprinted expression and parent-of origin for each gene may be located at <http://igc.otago.ac.nz/table.html>.

Reference List

1. Luo RZ, Peng H, Xu F, Bao J, Pang Y *et al.* (2001) Genomic structure and promoter characterization of an imprinted tumor suppressor gene ARHI. Biochim Biophys Acta 1519: 216-222.

2. Wood AJ, Roberts RG, Monk D, Moore GE, Schulz R *et al.* (2007) A screen for retrotransposed imprinted genes reveals an association between X chromosome homology and maternal germ-line methylation. PLoS Genet 3: e20.

3. Arima T, Yamasaki K, John RM, Kato K, Sakumi K *et al.* (2006) The human HYMAI/PLAGL1 differentially methylated region acts as an imprint control region in mice. Genomics 88: 650-658.

4. Arnaud P, Monk D, Hitchins M, Gordon E, Dean W *et al.* (2003) Conserved methylation imprints in the human and mouse GRB10 genes with divergent allelic expression suggests differential reading of the same mark. Hum Mol Genet 12: 1005-1019.

5. Ono R, Shiura H, Aburatani H, Kohda T, Kaneko-Ishino T *et al.* (2003) Identification of a large novel imprinted gene cluster on mouse proximal chromosome 6. Genome Res 13: 1696-1705.

6. Lefebvre L, Viville S, Barton SC, Ishino F, Surani MA (1997) Genomic structure and parent-of-origin-specific methylation of Peg1. Hum Mol Genet 6: 1907-1915.

7. Hancock AL, Brown KW, Moorwood K, Moon H, Holmgren C *et al.* (2007) A CTCF-binding silencer regulates the imprinted genes AWT1 and WT1-AS and exhibits sequential epigenetic defects during Wilms' tumourigenesis. Hum Mol Genet 16: 343-354.

8. Fitzpatrick GV, Soloway PD, Higgins MJ (2002) Regional loss of imprinting and growth deficiency in mice with a targeted deletion of KvDMR1. Nat Genet 32: 426-431.

9. Thorvaldsen JL, Duran KL, Bartolomei MS (1998) Deletion of the H19 differentially methylated domain results in loss of imprinted expression of H19 and Igf2. Genes Dev 12: 3693-3702.

10. Geuns E, De TN, Hilven P, Van SA, Liebaers I *et al.* (2007) Methylation analysis of the intergenic differentially methylated region of DLK1-GTL2 in human. Eur J Hum Genet 15: 352-361.

11. Kantor B, Shemer R, Razin A (2006) The Prader-Willi/Angelman imprinted domain and its control center. Cytogenet Genome Res 113: 300-305.

12. Szeto IY, Barton SC, Keverne EB, Surani AM (2004) Analysis of imprinted murine Peg3 locus in transgenic mice. Mamm Genome 15: 284-295.

13. Kelsey G, Bodle D, Miller HJ, Beechey CV, Coombes C *et al.* (1999) Identification of imprinted loci by methylation-sensitive representational difference analysis: application to mouse distal chromosome 2. Genomics 62: 129-138.

14. Peters J, Wroe SF, Wells CA, Miller HJ, Bodle D *et al.* (1999) A cluster of oppositely imprinted transcripts at the Gnas locus in the distal imprinting region of mouse chromosome 2. Proc Natl Acad Sci U S A 96: 3830-3835.

15. Williamson CM, Ball ST, Nottingham WT, Skinner JA, Plagge A *et al.* (2004) A cis-acting control region is required exclusively for the tissue-specific imprinting of Gnas. Nat Genet 36: 894-899.

16. Liu J, Yu S, Litman D, Chen W, Weinstein LS (2000) Identification of a methylation imprint mark within the mouse Gnas locus. Mol Cell Biol 20: 5808-5817.

17. Coombes C, Arnaud P, Gordon E, Dean W, Coar EA *et al.* (2003) Epigenetic properties and identification of an imprint mark in the Nesp-Gnasxl domain of the mouse Gnas imprinted locus. Mol Cell Biol 23: 5475-5488.
