# Supplementary material for: Evaluation of Allelic Expression of Imprinted Genes in Adult Human Blood
Source: PLoS One. 2010 Oct 21;5(10):e13556. doi: 10.1371/journal.pone.0013556 (PMC2958851; doi:10.1371/journal.pone.0013556)
Supplement: Table S2 — Quantitative RT-PCR primers for analysis of gene expression levels. PCR carried out using cDNA template, i.e. post reverse transcription reaction (RT-PCR). Gene transcripts, primers and amplicon size are shown. Each reaction was carried out with a Tm of 60°C for 40 cycles. DM = primers designed by Dave Monk, JF = primers designed by Jennifer Frost. (0.09 MB DOC) [file pone.0013556.s007.doc]

Table S2

| **Locus** | **Transcript** | **DIR^N^** | **5’-3’** | **DIR^N^** | **5’-3’** | **SIZE (BP)** | **ACCESSION** | **REF** |
| --- | --- | --- | --- | --- | --- | --- | --- | --- |
| 1p31 | *DIRAS3* | F1 | GTCTTCTAGGCTGCTTGGTTCG | R1 | GCAGCTTCTGTTCCTTGGAG | 140 | NM_004675 | JF |
| 1p36 | *TP73* | F2IN | GCAAGCGTGCCTTCAAGCAGAG | R2 | GGCCTCGCACCTGAAGGTAG | 108 | NM_005427 | JF |
| 4q22 | *NAP1L5* | F2 | Gtcatgtagcttttagggtg | R2 | ggcacagctaatacaagcaaac | 159 | NM_153757 | DM |
| 6q24 | PLAGL1 Iso 1 | NOX F | GGCATCTGCCATTTGTCATTCAG | NOX R | GTGTCTAAATCAAGGCTCGG | 58 | NM_001080954 | JF |
|  | PLAGL1 Iso 2 | F | GGCGGGTAGGTAGGAAAGG | COM  ROUT | GATGTTTATGAATCAGGCAGG | 52 | NM_001080951 | JF |
| 6q25 | *IGF2R* | F | GAAAACCCTGGGAACTCCTG | RIN | GCATGGCACCTCCTTATTTG | 118 | NM_000876 | JF |
| 7p11 | *GRB10* ALL ISO | FOUT | GCAAACAGGACGCGTGATAGAG | R | GTGAATCACTGTACTTAGGG | 148 | NM_001001555 | JF |
| 7q21 | *PEG10* | *1-2F* | AGGAGTCCTCGCGTGAAATAAG | ROUT | AGACCTCCCAGCTGTAGCTTCAC | 115 | NM_015068 | DM |
|  | *SGCE* | F | AAAATGTGGGGAAGCCAACAATC | R | CTGCTTGATATGGCAACGGGA | 126 | NM_003919 | JF |
|  | *MESTIT1* | *F* | ACTGAGGAAACTACCGCCTATAAG | R | ATGTGGGTAGACATGTTCCATG | 157 | NR_004382 | JF |
|  | *MEST* Iso 1 | F | GGCATGGGATAACGCGGCCATGG | R | GCAGGTACGCAGCAAGCAGG | 107 | NM_002402 | JF |
|  | *MEST* Iso 2 | F | GGCCGAGAACCTCTGGCCTCAGG |  |  | 146 | NM_177524 | JF |
| 10q26 | *INPP5F*  *_V2* | *F2* | GTTGACATTTTCCGACTGCCC | R2 | CTACTACTAATTACAGTGTTAC | 144 | NR_003252 | DM |
| 11p15 (TEL) | *H19* | *F* | GGAGTTGTGGAGACGGCCTTGAGT | R | CCAGTCACCCGGCCCAGATGGAG | 100 | NR_002196 | [1] |
|  | *IGF2* | *F* | TCGTTGAGGAGTGCTGTTTCC | R | ACACGTCCCTCTCGGACTTG | 87 | NM_000612 | [2] |
| 11p15 (CENT) | *PHLDA2* | *F* | GAGCGCACGGGCAAGTA | R | CAGCGGAAGTCGATCTCCTT | 68 | NM_003311 | [2] |
|  | *SLC22A18* | *Ex2 CUTF* | GCAGGATGAGCGCTCTAGGC | Ex3 R | GCACCCCGAAGGTGGTTTGC | 168 | NM_183233 | DM |
|  | *CDKN1C* | F | CAGCTGCACTCGGGGATTTC | 2R | GATCTCTTGCGCTTGGCGAAG | 125 | NM_000076 | DM |
|  | *KCNQ1OT1* | *F* | Tctctcttccaaatcataaatg | R | taaacaatgtcttgataaaggg | 194 | NM_000128 | JF |
|  | *KCNQ1* | F | GGATCGCGGCAGCAACACGATCGG | ROUT | GGTGCTGCCACCGTGCAAGG | 130 | NM_000218 | JF |
| 11p13 | *WT1* | F | CGCTATTCGCAATCAGGGTTAC | R | ATGGGATCCTCATGCTTGAATG | 142 | NM_024426 | [3] |
|  | *AWT1* | F | GAGAAG GTTACAGCACGGTCAC | R | ATGGGATCCTCATGCTTGAATG | 108 | AK093168 | DM |
|  | *WT1-AS (WIT1)* | INF | AGAGTCCGTTCAG GAATCCTTG | OUTR | AGGCTGCAGGGAACTCCTCCCA | 112 | NR_023920 | DM |
| 13q14 | *HTR2A* | *F2* | CCATCCAGAATCCCATCCACC | R2 | CCTTCGAATCGTCCTGTAGCCC | 95 | NM_000621 | JF |
| 14q32 | *MEG3* | FIN | GAACTTGAAGAGGTTTAGCCGG | ROUT | GGACCAGCCTTCCAAATGGG | 122 | AF090934 | JF |
|  | *DLK1* | *FEX2-3* | ATGACAATGTTTGCAGGTGCCAG | RIN | TGCCCGGGTTCTCCACAGAGTC | 93 | NM_003836 | DM |
|  | *DIO3* | FIN | CCACATGAACAATCTCCCCTACC | R | CCGCGCTCAAAAGAGGATTTCC | 103 | NM_001362 | JF |
| 15q11 | *NDN* | F | GCCCGAATACGAGTTCTTTT | R | GCCTCCTCCAGAGCTTCTCTGT | 138 | NM_002487 | JF |
|  | *SNRPN* | *F* | GTCTTCAGAAGCATCAAGTTTTAAC | R | GCCATCTTGCAGGATACATCTC | 127 | NM_022806 | JF |
|  | *IPW* | *F OUT* | CCTTGCAGAAGATGACTTCC | R | CAAATCCACCTCTTTGTGGCC | 149 | NR_023915 | JF |
| 19q13 | *PEG3* | *FOUT* | TCCAGGACAACATGGAAAACTACAG | ROUT | ATCACTCCGTGGGAAGATTC | 211 | NM_006210 | DM |
| 20q11 | *MCTS2* | F | GTCAGTTATTAAGGGCATTAAG | R | GTATCTACTGCAGCAGGGTACAG | 333 | BC053868 | DM |
| 20q11 | *NNAT* | FOUT | TCGGCTGGTACATCTTCCGCGT | ROUT | AGGGAGTACCTGAACACCTCA | 130 | NM_005386 | DM |
| 20q13 | *NESP* | *F* | AAGGGACCCATCCCCATCCGGC | GNAS R | ATGGTGCTTTTACCAGATTCTCC | 100 | NM_016592 | JF |
|  | *GNAS* | *F* | CAGCGCAACGAGGAGAAGGCGCA | R | ATGGTGCTTTTACCAGATTCTCC | 134 | NM_000516 | JF |
|  | *GNAS Exon 1A* | F2OUT2 | GCTCTCTGGCTCCGGGCTGCG | R2 | GGTGCTTTTACCAGATTCTCCAG | 140 | X56009 | JF |
|  | *GNAS XL* | FIN | TACGCACCGCCTGCTGCCTC | RIN | CCATTAACATGCAGGATCCTC | 84 | NM_001077490 | JF |

**Table S2. Quantitative RT-PCR primers** for analysis of gene expression levels. PCR carried out using cDNA template, i.e. post reverse transcription reaction (RT-PCR). Gene transcripts, primers and amplicon size are shown. Each reaction was carried out with a Tm of 60ºC for 40 cycles. DM = primers designed by Dave Monk, JF = primers designed by Jennifer Frost

Reference List

1. Gicquel C, Rossignol S, Cabrol S, Houang M, Steunou V *et al.* (2005) Epimutation of the telomeric imprinting center region on chromosome 11p15 in Silver-Russell syndrome. Nat Genet 37: 1003-1007.

2. Apostolidou S, bu-Amero S, O'Donoghue K, Frost J, Olafsdottir O *et al.* (2007) Elevated placental expression of the imprinted PHLDA2 gene is associated with low birth weight. J Mol Med 85: 379-387.

3. Siehl JM, Thiel E, Leben R, Reinwald M, Knauf W *et al.* (2002) Quantitative real-time RT-PCR detects elevated Wilms tumor gene (WT1) expression in autologous blood stem cell preparations (PBSCs) from acute myeloid leukemia (AML) patients indicating contamination with leukemic blasts. Bone Marrow Transplant 29: 379-381.
