# Supplementary material for: Evaluation of Allelic Expression of Imprinted Genes in Adult Human Blood
Source: PLoS One. 2010 Oct 21;5(10):e13556. doi: 10.1371/journal.pone.0013556 (PMC2958851; doi:10.1371/journal.pone.0013556)
Supplement: Table S3 — Allele specific assays for gene expression - detailing SNPs, transcripts, primers and amplicon size. Genotyping, PCR using genomic DNA template; RT, PCR using cDNA template, i.e. post reverse transcription reaction; D, direction; Tm, annealing temperature; JF, primers designed by Jennifer Frost. (0.14 MB DOC) [file pone.0013556.s008.doc]

**Table S3**

| *Gene* | | SNP | Genotyping | | | | RT | | | | | Accession Number | Reference |
| --- | --- | --- | --- | --- | --- | --- | --- | --- | --- | --- | --- | --- | --- |
|  |  | | Size (bp) | D | Primer 5’-3’ | | Size (bp) | | | D/  Isoform | Primer 5’-3’ |  |  |
| *NAP1L5* | | rs710834 | 389 | F | GCGGCTTCTCCTCTAACATG | | | | | | | [NM_153757](http://www.ncbi.nlm.nih.gov/entrez/query.fcgi?cmd=Search&db=Nucleotide&term=NM_153757&doptcmdl=GenBank&tool=genome.ucsc.edu) | [1] |
|  |  |  |  | R | GGTGAGCTCTTGGATCTTGG | | | | | | |  |  |
| *PLAGL1* | | rs2092894 | 203 | F | CAAGAGGACACGCTAAGAACG | | 662 | | | ISO1 F | AGCCGTGCTCACAGCTCAG | [NM_002656](http://www.ncbi.nlm.nih.gov/entrez/query.fcgi?cmd=Search&db=Nucleotide&term=NM_002656&doptcmdl=GenBank&tool=genome.ucsc.edu) | [2] |
|  |  |  |  |  |  |  | 589 | | | ISO2 F | CGGACTCCAGAACTTTCCAA | NM_001080951 |  |
|  |  |  |  | R | TGGTGGACCCTACCTCAGTT | | | | | | |  |  |
| *IGF2R* | | rs614754  rs1805075 | 222 | F | GTTGTCTGCCCTCCAAAGAA | | | | | | | NM_000876 | JF |
|  |  |  |  | R | CATAGTATTCCCACTTGAG | 283 | | | | R | ctttggagtacgtgacaac |  |  |
| *GRB10* | | rs1800504 | 160 | F | GTCAGACACGGTGCCCCTCCT |  | | | | | | NM_005311 | [3] |
|  | |  |  | R | ATAAGGCCTGGACCTACCTGACA | 166 | | | | R | CTGGTCTTCCTCCTGAAGGCGC |  |  |
| *PEG10* | | ACI1 | 352 | F | ACAGAGATGTAAGAGGCAGGC | | | | | | | NM_015068 | [4] |
|  | |  |  | R | ATTCACAGCATTGTAAGAAGTTCAC | | | | | | |  |  |
| *MEST* | | rs1050582 | 274 | F | GATGACCACATTAGCCACTATC | 1214 | | | | ISO1 F | ATGGGATAACGCGGCCATGGTG | NM_002402 | [5], JF |
|  | |  |  |  |  | 1203 | | | | ISO2 F | AGTCCTGTAGGCAAGGTCTTACCTG | NM_177524 |  |
|  | |  |  | R | CTATTATGTCAACTTAGTCAG | | | | | | |  |  |
| *INPP5F_V2* | | rs3188055 | 634 | F | GATTACTGTTGCTCATGGGAGTGG | 1438 | | | | F | TCCGACTGCCTGTTACGTGC | NR_003252 | [1] |
|  |  |  |  | R | CTGAATTATCCGTGTCTGGCATTG |  |  |  |  | R | GGGTTTTTTGAGATGCAACTGAATG |  |  |
| *SLC22A18* | | rs1048046  rs1048047 | 227 | F | CCCGGATCAACTGGACTTTTG | | | | | | | NM_183233 | [6] |
|  |  |  |  | R | GGCACGATGGAGAACTGCATG | 295 | | | | R | GCACCCCGAAGGTGGTTTGCA |  |  |
| *KCNQ1OT1* | | MSP1  rs231357 | 197 | F | GCTACATCTCTCTTCCAAATC | | | | | | | NM_000128 | [6] |
|  |  |  |  | R | ACAATGTCTTGATAAAGGGG | | | | | | |  |  |
|  |  | SAC1  rs231359 | 190 | F | CTTGAGAGAAACAATCCCACAG | | | | | | |  |  |
|  |  |  |  | R | GTATGGCTTTTCAGTGTTCC | | | | | | |  |  |
|  |  | rs10832514 | 277 | F | gaatcagatgccctcaatctg | | | | | | |  | [7] |
|  |  |  |  | R | CACAAGTTGGAGAGGGCTGAG | | | | | | |  |  |
| *KCNQ1* | | rs1057128 | 190 | F | ctgtcactgcctgcactttg | 271 | | F | | | CTTCGCCGAGGACCTGGACCTG | NM_000218 | [6] |
|  | |  |  | R | GCCGTTTGGCCGTGCCCAC |  |  | R | | | GGGAAGCCCTCACTGTTCATC |  |  |
| *IGF2* | | APA1 | 235 | F | CTTGGACTTTGAGTCAAATTGG | | | | | | | NM_000612 | [8] |
|  | |  |  | R | CTCCTTTGGTCTTACTGGG | | | | | | |  |  |
| *NDN* | | rs2192206 | 540 | F | gcccgaatacgagttctttt | | | | | | | NM_002487 | [9] |
|  | |  |  | R | cacacatcatcagtcccata | | | | | | |  |  |
| *SNRPN* | | rs705 | 423 | F | catcagtcctaagtgtgtc | 293 | | F | | | CAGGCATTCTTAGCTGAGAC | NM_022806 | JF |
|  |  |  |  | R | GATCACTGCACATGCTGGCAAAC |  | | R | | | CATCTTGCAGGATACATCTC |  |  |
| *IPW* | | rs691 | 396 | F | CTGCATGATTTTTTTTCAAAAA | | | | | | | NR_023915 | [10] |
|  | |  |  | R | ATATAGGGAGGTTCATTGCACA | | | | | | |  |  |
| *GNAS* ALL ISOFORMS | | rs7121 | 644 | F | gtcgggatgtctttatgaaag |  | | F | | | CTGCAAGGAGCAACAGCGATG |  | JF |
|  |  |  |  | R | CAGGGCTGTCACTCATGTTC |  | | R | | | GTCAATCAGCTGGTACTCGTTG |  |  |
| *GNAS* | |  |  |  | | 365 | | F | | | CCACGCACCGCCTGCTGCTG | NM_001077489 |  |
| *EXON 1A* | |  |  |  |  | 551 | | F | | | GGTTAGAAGCTCTGCTCCC | NR_003259 |  |
|  |  |  |  |  |  |  | | | R |  | CTGTGGGAGGATGAAGGAGTGC |  |  |
|  |  |  |  |  |  | 259 | | F | | + Exon3 | GAAGAGGACCCGCAGGCTGC | X56009 |  |
|  |  |  |  |  |  | 218 | | F | | – Exon3 | GAAGGCAACCAAAGTGCAGGACATC | CR590357 |  |

Allele specific assays for gene expression – detailing SNPs, transcripts, primers and amplicon size. Genotyping, PCR using genomic DNA template; RT, PCR using cDNA template, i.e. post reverse transcription reaction; D, direction; JF, primers designed by Jennifer Frost

Reference List

1. Wood AJ, Roberts RG, Monk D, Moore GE, Schulz R *et al.* (2007) A screen for retrotransposed imprinted genes reveals an association between X chromosome homology and maternal germ-line methylation. PLoS Genet 3: e20.

2. Valleley EM, Cordery SF, Bonthron DT (2007) Tissue-specific imprinting of the ZAC/PLAGL1 tumour suppressor gene results from variable utilization of monoallelic and biallelic promoters. Hum Mol Genet 16: 972-981.

3. McCann JA, Zheng H, Islam A, Goodyer CG, Polychronakos C (2001) Evidence against GRB10 as the gene responsible for Silver-Russell syndrome. Biochem Biophys Res Commun 286: 943-948.

4. Sun BW, Yang AC, Feng Y, Sun YJ, Zhu Y *et al.* (2006) Temporal and parental-specific expression of imprinted genes in a newly derived Chinese human embryonic stem cell line and embryoid bodies. Hum Mol Genet 15: 65-75.

5. McMinn J, Wei M, Sadovsky Y, Thaker HM, Tycko B (2006) Imprinting of PEG1/MEST isoform 2 in human placenta. Placenta 27: 119-126.

6. Monk D, Arnaud P, Apostolidou S, Hills FA, Kelsey G *et al.* (2006) Limited evolutionary conservation of imprinting in the human placenta. Proc Natl Acad Sci U S A 103: 6623-6628.

7. Weksberg R, Nishikawa J, Caluseriu O, Fei YL, Shuman C *et al.* (2001) Tumor development in the Beckwith-Wiedemann syndrome is associated with a variety of constitutional molecular 11p15 alterations including imprinting defects of KCNQ1OT1. Hum Mol Genet 10: 2989-3000.

8. Cui H, Horon IL, Ohlsson R, Hamilton SR, Feinberg AP (1998) Loss of imprinting in normal tissue of colorectal cancer patients with microsatellite instability. Nat Med 4: 1276-1280.

9. MacDonald HR, Wevrick R (1997) The necdin gene is deleted in Prader-Willi syndrome and is imprinted in human and mouse. Hum Mol Genet 6: 1873-1878.

10. Kim KP, Thurston A, Mummery C, Ward-van OD, Priddle H *et al.* (2007) Gene-specific vulnerability to imprinting variability in human embryonic stem cell lines. Genome Res 17: 1731-1742.
