# Supplementary material for: Evaluation of Allelic Expression of Imprinted Genes in Adult Human Blood
Source: PLoS One. 2010 Oct 21;5(10):e13556. doi: 10.1371/journal.pone.0013556 (PMC2958851; doi:10.1371/journal.pone.0013556)
Supplement: Table S4 — Bisulphite PCR primers for analysis of differential methylation at imprinted differentially methylated regions. Amplicons were subjected to Combined Bisulphite and Restriction (CoBRA) assays and bisulphite sequencing. Primers are specific for bisulphite converted DNA. Where CpG dinucleotides appear in the primer, 1:1 primer mixes are prepared with either a C or a T base in the forwards direction (YG) and a G or an A base in the reverse (CR). Bisulphite PCR was carried out using Tm of 53 °C, except for KvDMR where the Tm was 50 °C, and all for 45 Cycles. The size/enzyme column indicates the restriction endonuclease used in the CoBRA assays. DM = primers designed by Dave Monk, JF = primers designed by Jennifer Frost. (0.09 MB DOC) [file pone.0013556.s009.doc]

Table S4

| **Locus** | **CpG Island** | **Foward 5’-3’** | **Reverse 5’-3’** | **Size (bp)**  **/enzyme** | **Accession** | **Ref** |
| --- | --- | --- | --- | --- | --- | --- |
| 6q24 | *PLAGL1* | GTGGATTTTATATTAGATAGG | CRCTTCCCCRAATCRCCRTC | 400/ 2x Tai1 | AL109755 | [1] |
| 7p11 | *GRB10* | GTYGGGGTTTTTGTTAGTTTG | CCAATCCCTCRAAAACTAAA | 518 MboI | AC004920 | [2] |
| 7q21 | *PEG10/SGCE* | GTGTTATGTTTTATAAATAGATAAG | AACTCATATACCTCTACAATTC | 376 Taqa1 | Chr7:94122795-94124463 | DM |
|  | *MEST* | TYGTTGTTGGTTAGTTTTGTAYGGTT | AAAAATAACACCCCCTCCTCAAAT | 289 Taqa1 | Chr7:129917976-129920347 | [3] |
|  |  |  | CCCAAAAACAACCCCAACTC |  |  |  |
| 11p15 (TEL) | *H19 DMD* | TGTTGAAGGTTGGGGAGATAGA | CCCAAACCATAACACTAAAACCCTC | 450 BstUI | Chr11:1977500-1979000 | [4] |
|  | *H19 PROM* | GGTATGGTGTTTTTTGAGGGGAGAT | CATCCCACCCCCTCCCTCACCCTA | 323 Tai1 | Chr11:1975373-1976765 | [5] |
|  | *IGF2 DMR0* | TTGGTGTTGGAAAGTGTTTG | CTATAACRTCCAAACCCTCTA | 300 Taqa1 | NM_001007139 | [6] |
|  | *KvDMR* | TGTTGAGGAGTTTYGGGGAGGATTA | CACCTCACACCCAACCAATACCTCAT | 372 Taqa1 | Chr11:2676987-2678663 | [7] |
| 14q32 | *IG-DMR* | GGGTTGGGTTTTGTTAGTTGTT | CCAATTACAATACCACAAAATTAC | 259 Taqa1 | Chr14:100345000-100348000 | [8] |
|  | *MEG3 PROM* | GTAAGTTTTATAGGTTGTAAAGGGGGTGTT | CCACAACTAATAACTAAAAAAATAAACATT | 216 Taqa1 |  |  |
| 15q11 | *SNURF/SNRPN* | GGTTTTTTTTTATTGTAATAGTGTTGTGGGG | CTCCAAAACAAAAAACTTTAAAACCCAAA | 408 Tai1 | Chr15:22751129-22752147 | [9] |
| 19q13 | *PEG3* | OUT –  GATGGTATTTAATGGGTGGGGTTGGAATAG | CTATCCTACCTATAAAAATTTTCTTTC | 343 Tai1 | Chr19:62043096-62044096 | DM |
|  |  | IN - GTTGGAATAGATTATTATATTTAATG |  |  |  |  |
| 20q13 | *NESP55* | OUT - GAGGATAAAGATTTAAGGGATTT | OUT - CTCAAACTCCCCAATTTAAC | 224 Tai1 | Chr20:56848531-56850548 | [10] |
|  |  | IN -  GAAGGAGTTTAAGGAGGAGAAGTAG | IN - CCATAAAAACAAAAAAAATCTAAAC |  |  |  |
|  | *EXON1A* | OUT - GTCGTTTTTGCGGTTGTTGAG | OUT - CCAACGAAAACATCCGAAAATCCC | 351 Tai1 | AL121917 | JF |
|  |  | IN - GGTAGTTTATGTCGTTTTAGTTG | IN - CTACTAACCACCCCTTCATC |  |  |  |
|  | *GNAS XL* | OUT –  GTGCGGTTCGTTTTATTTTTGTCGCGAACGC | ACTACTTCCTCCTCAACTAAAAATCTCTC | 291 Taqa1 | Chr20:56862420-56864634 | JF |
|  |  | IN - GTTATCGGTAGTTTATTTTAAGAGGTTG |  |  |  |  |

**Table S4 Bisulphite PCR primers** for analysis of differential methylation at imprinted differentially methylated regions. Amplicons were subjected to Combined Bisulphite and Restriction (CoBRA) assays and bisulphite sequencing. Primers are specific for bisulphite converted DNA. Where CpG dinucleotides appear in the primer, 1:1 primer mixes are prepared with either a C or a T base in the forwards direction (YG) and a G or an A base in the reverse (CR). Bisulphite PCR was carried out using Tm of 53 ºC, except for KvDMR where the Tm was 50 ºC, and all for 45 Cycles. The size/enzyme column indicates the restriction endonuclease used in the CoBRA assays. DM = primers designed by Dave Monk, JF = primers designed by Jennifer Frost

Reference List

1. Kamiya M, Judson H, Okazaki Y, Kusakabe M, Muramatsu M *et al.* (2000) The cell cycle control gene ZAC/PLAGL1 is imprinted--a strong candidate gene for transient neonatal diabetes. Hum Mol Genet 9: 453-460.

2. Monk D, Smith R, Arnaud P, Preece MA, Stanier P *et al.* (2003) Imprinted methylation profiles for proximal mouse chromosomes 11 and 7 as revealed by methylation-sensitive representational difference analysis. Mamm Genome 14: 805-816.

3. Kerjean A, Dupont JM, Vasseur C, Le TD, Cuisset L *et al.* (2000) Establishment of the paternal methylation imprint of the human H19 and MEST/PEG1 genes during spermatogenesis. Hum Mol Genet 9: 2183-2187.

4. Vu TH, Li T, Nguyen D, Nguyen BT, Yao XM *et al.* (2000) Symmetric and asymmetric DNA methylation in the human IGF2-H19 imprinted region. Genomics 64: 132-143.

5. Frevel MA, Sowerby SJ, Petersen GB, Reeve AE (1999) Methylation sequencing analysis refines the region of H19 epimutation in Wilms tumor. J Biol Chem 274: 29331-29340.

6. Monk D, Sanches R, Arnaud P, Apostolidou S, Hills FA *et al.* (2006) Imprinting of IGF2 P0 transcript and novel alternatively spliced INS-IGF2 isoforms show differences between mouse and human. Hum Mol Genet 15: 1259-1269.

7. Monk D, Arnaud P, Apostolidou S, Hills FA, Kelsey G *et al.* (2006) Limited evolutionary conservation of imprinting in the human placenta. Proc Natl Acad Sci U S A 103: 6623-6628.

8. Kawakami T, Chano T, Minami K, Okabe H, Okada Y *et al.* (2006) Imprinted DLK1 is a putative tumor suppressor gene and inactivated by epimutation at the region upstream of GTL2 in human renal cell carcinoma. Hum Mol Genet 15: 821-830.

9. Zeschnigk M, Schmitz B, Dittrich B, Buiting K, Horsthemke B *et al.* (1997) Imprinted segments in the human genome: different DNA methylation patterns in the Prader-Willi/Angelman syndrome region as determined by the genomic sequencing method. Hum Mol Genet 6: 387-395.

10. Bastepe M, Frohlich LF, Hendy GN, Indridason OS, Josse RG *et al.* (2003) Autosomal dominant pseudohypoparathyroidism type Ib is associated with a heterozygous microdeletion that likely disrupts a putative imprinting control element of GNAS. J Clin Invest 112: 1255-1263.
